# Qualitative Chemical Characterization and Multidirectional Biological Investigation of Leaves and Bark Extracts of *Anogeissus leiocarpus* (DC.) Guill. & Perr. (Combretaceae)

**DOI:** 10.3390/antiox8090343

**Published:** 2019-09-01

**Authors:** Giustino Orlando, Claudio Ferrante, Gokhan Zengin, Kouadio Ibrahime Sinan, Kouadio Bene, Alina Diuzheva, József Jekő, Zoltán Cziáky, Simonetta Di Simone, Lucia Recinella, Annalisa Chiavaroli, Sheila Leone, Luigi Brunetti, Carene Marie Nancy Picot-Allain, Mohamad Fawzi Mahomoodally, Luigi Menghini

**Affiliations:** 1 Department of Pharmacy, University “G. d’Annunzio” of Chieti-Pescara, 66100 Chieti, Italy; 2 Department of Biology, Faculty of Science, Selcuk University, Konya 42130, Turkey; 3 Laboratoire de Botanique et Phytothérapie, Unité de Formation et de Recherche Sciences de la Nature, Université Nangui Abrogoua, 02 BP 801 Abidjan 02, Ivory Coast; 4 Department of Forest Protection and Entomology, Faculty of Forestry and Wood Sciences, Czech University of Life Sciences, 16500 Prague, Czech Republic; 5 Agricultural and Molecular Research and Service Institute, University of Nyíregyháza, 4400 Nyíregyháza, Hungary; 6 Department of Health Sciences, Faculty of Science, University of Mauritius, 230 Réduit, Mauritius

**Keywords:** *Anogeissus*, bioactive compounds, antioxidant, enzyme inhibition, ulcerative colitis

## Abstract

*Anogeissus leiocarpus* (DC.) Guill. & Perr. (Combretaceae) has a long history of use by folk populations for the management of multiple human ailments. Based on the published literature, there has been no attempt to conduct a comparative assessment of the biological activity and the phytochemical profiles of the leaves and stem bark of *A. leiocarpus* extracted using methanol, ethyl acetate, and water. By high-performance liquid chromatography with electrospray ionization mass spectrometric detection (HPLC-ESI-MS^n^) analysis, quinic, shikimic, gallic, and protocatechuic acids were tentatively identified from all the extracts, while chlorogenic, caffeic, ferulic, and dodecanedioic acids were only characterised from the leaves extracts. Additionally, a pharmacological study was carried out to evaluate potential protective effects that are induced by the extracts in rat colon and colon cancer HCT116 cell line. In general, the methanol and water extracts of *A. leiocarpus* leaves and stem bark showed potent radical scavenging and reducing properties. It was noted that the stem bark extracts were more potent antioxidants as compared to the leaves extracts. The methanol extract of *A. leiocarpus* leaves showed the highest acetyl (4.68 mg galantamine equivalent/g) and butyryl (4.0 mg galantamine equivalent/g) cholinesterase inhibition. Among ethyl acetate extracts, the pharmacological investigation suggested stem bark ethyl acetate extracts to be the most promising. This extract revealed ability to protect rat colon from lipopolysaccharide-induced oxidative stress, without exerting promoting effects on HCT116 cell line viability and migration. As a conclusion, *A. leiocarpus* represents a potential source of bioactive compounds in the development of novel therapeutic agents.

## 1. Introduction

*Anogeissus leiocarpus* (DC.) Guill. & Perr. (Combretaceae), also known as chewing stick or axlewood tree, has a long history of traditional use for the management of multiple human ailments. The leaves of *A. leiocarpus* are used in the treatment of skin diseases, fever, diarrhoea, malaria, and stomach infections [1]. *A. leiocarpus* is used by the Yoruba people in Nigeria to treat bacterial infections and the roots and twigs of the plant are used as chewing sticks for dental hygiene. Various parts of the plant (roots, leaves, stem bark, and twigs) are used in the management of gonorrhoea, cough, wounds, acute respiratory tract infections, stomach infections, fever, tuberculosis, dysentery, giardiasis, malaria, trypanosomiasis, yellow fever, jaundice, and pathogenic microbial infections [2]. The water extract of *A. leiocarpus* stem bark was recently found to combat erectile dysfunction in paroxetine-induced sexually impaired male Wistar rats [3]. A spontaneous decrease in serum glucose level in alloxan-induced diabetic rats administered with the aqueous extract of *A. leiocarpus* leaves [4] was linked to the α-amylase and α-glucosidase inhibitory action of the extract [5]. The aqueous extract of *A. leiocarpus* trunk bark was reported to exert significant antihypertensive effects in NG-nitro-L-arginine methyl ester (L-NAME)-induced hypertensive rats [6]. The methanolic and ethyl acetate extracts of *A. leiocarpus* leaves exhibited antioxidant and antibacterial properties [7]. The stem bark methanolic extract of *A. leiocarpus* demonstrated antitrypanosomal activity against four *Trypanosoma* strains [8] and leishmanicidal activity [9]. The methylene chloride extract of *A. leiocarpus* (IC_50_ value of 3.8 μg/mL) showed in vitro antiplasmodial activity against *Plasmodium falciparum*, the protozoan parasite that is responsible for malaria in human [10]. Lately, a group of researchers investigated the effect of *A. leiocarpus* methanolic extract on the liver function in mice that were infected with *Plasmodium berghei* [11].

From the literature, several studies attempted to investigate the biological activity, mainly, the antibacterial properties, of different extracts of *A. leiocarpus*. However, as far as our literature search could ascertain, no study was focused on the comparative evaluation of the phytochemical profiles of the methanol, ethyl acetate, and water extracts of the leaves and stem bark of *A. leiocarpus*. Additionally, in the present study, the authors present the antioxidant and inhibitory action of *A. leiocarpus* extracts on key enzymes that are related to diabetes type II, Alzheimer’s disease, and skin hyperpigmentation. Finally, while considering the potential antiproliferative effects that are exerted by *A. latofolia* on colon cancer cells [12], the antiproliferative effects of *A. leiocarpus* extracts were tested on human colon cancer HCT116 cell line. Additionally, the same extracts were tested for their putative antioxidant/anti-inflammatory effects on isolated rat colon specimens that were exposed to *E. coli* lipopolysaccharide (LPS), in order to reproduce the burden of oxidative stress and inflammation occurring in ulcerative colitis [13]. To this regard, selected biomarkers of oxidative stress/inflammation, including prostaglandin (PG)E_2_, 8-iso-PGF_2α_, and serotonin (5-HT) were selected. It is expected that detailed phytochemical profiles of the different extracts will enable tentative identification of phytochemical/s, which might be responsible for the observed biological activity.

## 2. Materials and Methods

### 2.1. Plant Material and Preparation of Extracts

The sampling of the plant species was done in Gontougo region (Sandegue) of Ivory Coast in the year 2018. Botanical authentication of the plant was done by the botanist Dr. Kouadio Bene (Laboratoire de Botanique et Phytothérapie, Université Nangui Abrogoua, Abidjan, Ivory Coast). The leaves and stem barks were dried at room temperature (in shade, about 10 days). These materials were then powdered by using a laboratory mill.

Methanol and ethyl acetate extracts were prepared through maceration techniques (five grams of plant samples were mixed with one hundred ml of each solvents for 24 h). After maceration, the extracts were subjected to filtration and evaporation in vacuo at 40 °C. Traditional infusion was selected to prepare the water extract (five grams of plant samples were infused with one hundred mL of boiling water for 20 min.). After preparation, the water extract was subjected to filtration and freeze drying. Finally, the extracts were stored at 4 °C until phytochemical and pharmacological analysis.

### 2.2. Profile of Bioactive Compounds

Total phenols, flavonoids, phenolic acids, and flavonols were assayed through spectrophotometric assays [14,15], The extract concentrations of phenolics, flavonoids, phenolic acids, flavonols and tannins, and saponins were determined through spectrophotometric assays [14,15], and were expressed as equivalents of gallic acid (mg GAE/g dry extract), rutin (mg RE/g dry extract), caffeic acid (mg CAE/g dry extract), catechin (mg CE/g dry extract), and quillaja (mg QE/g dry extract), respectively.

The qualitative analysis of *A. santonicum* extracts (5 mg/mL) was carried out according the protocol that was described by Zengin et al. [16].

An high performance liquid chromatography (HPLC)-fluorimetric analysis was carried out in order to quantify the selected phenolic compounds, in *A. santonicum* extracts (5 µg/mL). To this regard, an HPLC apparatus (MOD. 1525, Waters Corporation, Milford, MA, USA) coupled to fluorimetric detector (MOD. 2475, Waters Corporation, Milford, MA, USA) and a C18 reversed-phase column (Phenomenex Kinetex, Torrance, CA, USA, 150 mm × 4.6 mm i.d., 2.6 µm) were used. The HPLC gradient conditions were selected, as previously mentioned by Rodrıguez-Delgado and coworkers [17]. In agreement with the same authors, λex = 278 nm and λem = 360 nm were selected in order to analyze the following phenolic compounds: gallic acid, catechin, and epicatechin.

### 2.3. Determination of Antioxidant and Enzyme Inhibitory Effects

The evaluation of anti-α-amylase, anti-α-glucosidase, anti-cholinesterases, and anti-tyrosinase activities was carried out as previously described by Uysal and coworkers [18]. The enzyme inhibitory results were evaluated in terms of standard equivalents; galatamine for cholinesterase (mg GALAE/g dry extract); kojic acid for tyrosinase (mg KAE/g dry extract), acarbose for amylase, and glucosidase (mmol ACAE/g dry extract). According to the same paper [18], the antiradical activity of the extracts was measured through the use of ferric reducing antioxidant power (FRAP), 2,2’-azino-bis(3-ethylbenzothiazoline-6-sulphonic acid) (ABTS) cupric reducing antioxidant capacity (CUPRAC), 2,2-diphenyl-1-picrylhydrazyl (DPPH), phosphomolydenum, and metal chelating tests. The antioxidant results were explained as equivalents of trolox (mg TE/g dry extract) and ethylenediaminetetraacetic acid (EDTA) (in metal chelating assay) (mg EDTAE/g dry extract). One-way ANOVA, followed by Tukey’s post hoc test, were applied for comparing the samples in terms of bioactive compounds content and biological activities. The MCA (multiple correspondence analysis) and Clustering Image Map were performed for the discrimination between the samples based on their chemical compositions and Venn graph was built to identify the chemical profile differences among those samples. Before, the data of the chemical composition were attributed to classes with two modalities (e.g., + for presence and – for the absence of compounds in extracts). Afterwards, Multiple datasets supervised analysis, namely DIABLO, was achieved to find out the key factor (parts and solvents) that is responsible for variation in datasets. Subsequently, the correlation between the bioactive compounds and biological activities were estimated. All of the statistical tests were conducted by using R 3.5.1 software environment.

### 2.4. Pharmacological Assays

#### 2.4.1. Allelopathy Assay

As previously described [19], the allelopathy bioassay was carried out in 90 mm diameter Petri dishes, which represented the substrate for the germination of seeds, whereas *A. leiocarpus* extracts (0.1–10 mg/mL) were dissolved in imbibition water. During the incubation period (three days at 4 °C), seeds were monitored in order to evaluate their uniform size and integrity. To this regard, lettuce could be considered as one of the most suitable dicotyledon for allelopathy assay. This is due to both fast germination rate and high sensitivity. After the third day of treatment, a root length ≥ 1 mm was the condition to consider positive the germination of seeds [19]. The experiments were carried out in triplicate and means ± SEM were determined through the use of GraphPad Prism software (version 5.01).

#### 2.4.2. Brine Shrimp Lethality Assay

*Artemia salina* lethality bioassay was performed, as previously reported [13]. The larvae of brine shrimps were exposed to the extracts (0.01–10 mg/mL) at 25–28 °C for 24 h. At the end of the incubation period, brine shrimp lethality was evaluated with the equation ((T – S)/T) × 100, being T and S the total number of larvae that were exposed to extracts and living nauplii, respectively. The experiments were carried out in triplicate.

### 2.5. In Vitro Studies

HCT116 cell line (ATCC^®^ CCL-247™) culture and differentiation were carried out as previously described in our published paper [13]. To evaluate the biocompatibility of *A. leiocarpus* extracts (0.1 mg/mL), the 3-(4,5-dimethylthiazol-2-yl)-2,5-diphenyltetrazolium bromide (MTT) viability test was carried out, as recently described [13]. The effects of extracts (0.1 mg/mL) on HCT116 cell viability was evaluated in comparison to the untreated control group 24 h after treatment. Finally, the effects of extracts on HCT116 cell spontaneous migration, through the use of wound healing test, as recently reported. Briefly, the cells were challenged with *A. leiocarpus* extracts (0.1 mg/mL), and spontaneous migration was monitored at different time points (0 and 24 h). The Image-J software (NIH) was used to quantify the scratch area, whereas GraphPad software was employed to calculate mean data at 0 and 24 h and express them as percentage variation with reference to relative 100% of that at 0 h.

### 2.6. Ex Vivo Studies

Male adult Sprague-Dawley rats (200–250 g) were sacrificed by CO_2_ inhalation, and colon specimens were immediately stimulated with *Escherichia coli* lipopolysaccharide (LPS) 10 µg/mL for 4 h (incubation period), as recently described [13]. Italian Health Minstry (authorization N. F4738.N.XTQ, delivered on 11th Novembre 2018) approved the experimental procedures.

During the incubation period, the colon specimens were also treated with *A. leiocarpus* extracts (0.1 mg/mL). Subsequently, extraction and chromatographic quantification of 5-HT (ng/mg wet tissue) was carried out in colon homogenate, as previously reported [20,21]. Additionally, colon homogenate was assayed for measuring PGE_2_ and 8-iso-PGF_2α_ via radioimmunoassay [22,23].

### 2.7. Statistical Analysis

Data were means ± SEM and analyzed by one-way analysis of variance (ANOVA), followed by Newman-Keuls post hoc test (GraphPad Prism version 5.01 for Windows, GraphPad Software, San Diego, CA, USA). The data were considered to be significant for *p* values less than 0.05. With the aim to apply 3Rs (Reduction, Refinement and Reduction) approach to the ex vivo procedures, the number of animals was determined through the “Resource Equation” N = (E + T)/T (10 ≤ E ≤ 20) [24]), where N, T, and E represent the number of animals, pharmacological treatments, and degree freedom in ANOVA, respectively.

## 3. Results

### 3.1. Phytochemical Profile

Table 1 presents phytochemical evaluations of the different extracts of *A. leiocarpus* leaves and stem bark. Quantitative determination showed that the stem barks extracts of *A. leiocarpus* (water extract = methanol extract > ethyl acetate extract) possessed significant amounts of phenolics when compared to their respective leaves extracts. The water extract of *A. leiocarpus* leaves showed the highest flavonoid (89.0 mg RE/g) and phenolic acid (14 mg CAE/g) contents. Highest tannin (77.0 mg CE/g), flavanol (79 mg CE/g), and saponin (438 mg QE/g) contents were recorded from the methanol extract of *A. leiocarpus* leaves. Phenolic acids, such as, protocatechuic acid, chlorogenic acid, caffeic acid, and ferulic acid, were previously reported to be soluble in polar protic solvents, like methanol, except gallic acid, which was readily soluble in water [25]. From Table 1, it is noted that no phenolic acid was recorded in the ethyl acetate extracts of *A. leiocarpus* leaves and stem bark. Caffeic acid, a phenolic acid, was found to be minimally soluble in ethyl acetate [26].

HPLC-fluorimeter analysis that was performed on selected phenolic compounds (i.e., gallic acid, catechin and epicatechin), revealed that leaf extract could have, overall, a higher content of these metabolites than stem bark extract (Table 2). This is consistent with the observed content of the total phenolic acids and flavanols.

Detailed analysis of the chemical composition of the ethyl acetate, methanol, and water extracts of *A. leiocarpus* leaves and stem bark by HPLC-ESI-MS^n^, while using both positive and negative ionisation modes, was also conducted. The detailed results are given as supplemental materials (Tables S1–S6). The results are also summarized in Table 3.

### 3.2. Phenolic Acids

Quinic ([M – H]^–^ at *m*/*z* 191), shikimic ([M – H]^–^ at *m*/*z* 173), gallic ([M – H]^–^ at *m*/*z* 169), and protocatechuic ([M – H]^–^ at *m*/*z* 153) acids were tentatively identified from all the extracts of *A. leiocarpus* leaves and stem bark. Chlorogenic ([M – H]^+^ at *m*/*z* 355), caffeic ([M – H]^–^ at *m*/*z* 179), ferulic ([M – H]^–^ at *m*/*z* 193), and dodecanedioic ([M – H]^–^ at *m*/*z* 229) acids were tentatively identified from the leaves extracts of *A. leiocarpus* only.

### 3.3. Flavonoids

Several compounds belonging to the flavonoid family were identified from *A. leiocarpus* extracts. The compound suffering deprotonation at *m*/*z* 269 [M – H]^–^ and fragment ions at *m*/*z* 225, 151, 149, and 117 was characterised as apigenin and was present in the methanol extract of *A. leiocarpus* leaves and water extract of *A. leiocarpus* stem bark. C-glucosides of apigenin ([M – H]^+^ at *m*/*z* 433), namely, vitexin and isovitexin, only occurred in the stem bark extracts of *A. leiocarpus.* Luteolin, suffering deprotonation at *m*/*z* 285 [M – H]^–^ and fragment ions at *m*/*z* 217, 199, 175, 151, and 133 was tentatively characterised. Other flavonoids, such as, catechin ([M – H]^–^ at *m*/*z* 289), naringenin ([M – H]^–^ at *m*/*z* 271), myricetin ([M – H]^–^ at *m*/*z* 317), taxifolin ([M – H]^–^ at *m*/*z* 303), and pinocembrin ([M – H]^–^ at *m*/*z* 255) were also identified.

### 3.4. Tannins Derivatives

Some tannin derivatives were tentatively characterised. Casuarinin with deprotonation at *m*/*z* 935 [M – H]^–^ and fragment ions at *m*/*z* 917, 783, 633, 300, and 275, was tentatively characterized in all the extracts. Hydrolysable tannins, namely chebulagic acid and punicalagin [27], were characterized at [M – H]^–^ *m*/*z* 953 and 1083, respectively. While punicalagin was identified in all of the extracts, chebulagic acid was only characterised in the leaves extracts.

### 3.5. Antioxidant Activities

Phytochemicals, which are ubiquitously present in plants, have been identified to possess antioxidant activity and they are capable of managing oxidative stress related diseases [28]. In this study, three types of antioxidant mechanisms were used, namely, radical scavenging, reducing power, and metal chelating. Table 4 presents the ability of *A. leiocarpus* extracts to scavenge DPPH and ABTS radicals. DPPH, which is a stable radical, is widely used to assess the free radical scavenging abilities of plant extract. By proton transfer, there is the DPPH change in the non-radical form, characterized by a yellow chromophore [29]. On the other side, the ABTS method is based on the monitoring of electron or hydrogen transfer-induced ABTS radical-cation decay, which is characterized by the disappearance of the corresponding blue-green radical [30]. From Table 4, it is observed that, in general, the methanol and water extracts of *A. leiocarpus* leaves and stem bark showed potent radical scavenging properties compared to the ethyl acetate extracts of *A. leiocarpus* leaves and stem bark. Besides, it was noted that the stem bark extracts were more potent radical scavengers when compared to the leaves extracts. Furthermore, the strong radical scavenging activities of the extracts was related to their high phenolic contents. Reducing power, as described by electron transfer ability, is considered to be one of the key indicators of antioxidant capacity of plant extracts [28]. In this study, the reducing power of *A. leiocarpus* extracts was assessed using FRAP and CUPRAC assays, which are characterized by the reduction of Fe^3+^ to Fe^2+^ and Cu^2+^ to Cu^+^, respectively [30,31]. Comparable to the free radical scavenging assessment, the methanol and water extracts of *A. leiocarpus* showed potent reducing properties as compared to the ethyl acetate extracts. The total antioxidant capacity that was determined by the phosphomolybdenum assay demonstrated that methanol and water extracts of *A. leiocarpus* leaves and stem bark were more potent antioxidants as compared to the ethyl acetate extracts. This finding is in line with radical scavenging and reducing power evaluations. The ability of *A. leiocarpus* extracts to chelate metal was also evaluated and is presented in Table 4. Given the recognised role of iron in oxidative stress, which is understood as an increase in oxygen radical intermediates concentration, eventually leading to dysregulation, the development of metal chelators having the ability to restore metal homeostasis and oxidative status appears to be a valuable challenge, particularly if the chelators possess other important biological activities that might mitigate other diseases [32]. Data that were gathered from this study revealed that the water extract of *A. leiocarpus* leaves, possessing the highest flavonoid content, was the most activity metal chelator. It has previously been proposed that flavonoids were potent chelators of iron [33]. Naringenin, quercetin, luteolin, and catechin compounds, belonging to the flavonoid family, were identified in the water extract of *A. leiocarpus* leaves and they were reported to possess metal chelating abilities [33,34]. For instance, quercetin was reported to form different complexes with Fe^2+^ through its 5-OH and 4-carbonyl groups [35].

### 3.6. Enzyme Inhibitory Activities

While the antioxidant activity of plant extract is often linked to the phenolic content, the enzyme inhibitory properties of extracts mainly involves the interaction of phytochemicals with the enzyme or enzyme-substrate complex. To the best of our knowledge, this is the first report on the assessment of the inhibitory potential of *A. leiocarpus* leaves and stem bark on enzymes related to Alzheimer’s disease and skin hyperpigmentation. A previous study has appraised the amylase and glucosidase inhibitory action of *A. leiocarpus* leaves [5]. However, no comparison has been made with the stem bark extract of the plant and the possible effect of different extraction solvents. Among the five food drug administration (FDA)-approved Alzheimer’s disease treatments, four are acetyl cholinesterase inhibitors [36]. Cholinesterase inhibitors designed for the management of Alzheimer’s disease stem from the cholinergic hypothesis, which is the leading theory proposed to explain the pathogenesis of Alzheimer’s disease [37]. It has been recognised that cholinergic neurons loss in brain area that is responsible for cognition and behaviour was the hallmark of Alzheimer’s disease. While the role of acetyl cholinesterase has been clearly claimed, the exact mechanism that involves butyryl cholinesterase remains elusive. Butyryl cholinesterase, previously underestimated in the pathogenesis of Alzheimer’s disease, was found to be up-regulated in advanced stages of the condition and plays a key role in the disease maintenance and progression [38]. From this perspective, it can be stated that cholinesterase inhibitors targeting both acetyl and butyryl cholinesterases are in need. Table 5 reports the acetyl cholinesterase inhibitory activity of the different extracts of *A. leiocarpus* leaves and stem bark ranging from 3.51 to 4.68 mg GALAE/g. With regards to the butyryl cholinesterase inhibitory action, the values ranged from 0.45 to 4.0 mg GALAE/g. Interestingly, the water extract of *A. leiocarpus* leaves only inhibited acetyl cholinesterase. It can be suggested that this extract might be targeted at the initial stage of the disease, when butyryl cholinesterase activity is not pronounced. The methanol extract of *A. leiocarpus* leaves (4.68 and 4.0 mg GALAE/g) showed potent inhibition against both cholinesterases. Over the past decades, there has been an emerging trend of naturally derived cosmetic products. This shift has encouraged researchers to find new cosmeceuticals and the focus has geared towards plants. Plant extracts have witnessed increased global demand for de-pigmenting agents due to their safety and compatibility with all skin types [39]. The inhibitory action of *A. leiocarpus* extracts on tyrosinase, a copper-containing enzyme responsible for the biosynthesis of melanin [40], was investigated. The data collected showed potent tyrosinase inhibition with values ranging from 113.0 to 155.26 mg KAE/g, the highest values was recorded for methanol extract of *A. leiocarpus* stem bark. Pinocembrin, shikimic acid, and vitexin, tentatively identified in the methanol extract of *A. leiocarpus* stem bark, were previously reported to inhibit tyrosinase [41,42,43]. A group of researchers [5] have reported the amylase (IC_50_ value of 242.17 μg/mL) and glucosidase (IC_50_ value of 196.35 μg/mL) inhibitory activity of *A. leiocarpus* leaves water extract. In the present investigation and, as opposed to the previous study, low inhibition was recorded against amylase, while no inhibitory action was observed against glucosidase in the presence of the water extract of *A. leiocarpus* leaves. The different activity that was recorded in our study might be related to the geographical location along with the environmental conditions of the studied *A. leiocarpus* plants. In this study, it was observed that the different *A. leiocarpus* extracts were poor inhibitors of amylase, with values that ranged from 0.19 to 1.13 mmol ACAE/g. Only ethyl acetate extracts inhibited glucosidase and the values were higher as compared to amylase. The inhibition of glucosidase is considered as strategic in the management of diabetes type II. Indeed, it has been advocated that the inhibition of glucosidase reduced post-prandial glucose rise and it was associated to less side effects.

### 3.7. Multivariate Analysis

**Figure 1:**
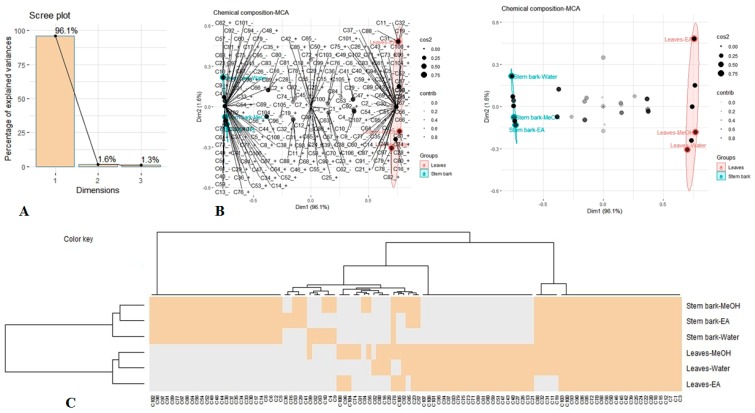
Multivariate analysis using multiple correspondence analysis (MCA) and Heatmap clustering analysis of chemical composition in *A. leiocarpus* extracts. (**A**): Percentage of explained variance per component. (**B**): projection of extracts and chemical compounds into the subspace spanned by the first two components of MCA. (**C**): Clustered Image Map (Euclidean Distance, Ward linkage). Gray colour: Absence, Wheat colour: Presence.

**Figure 2:**
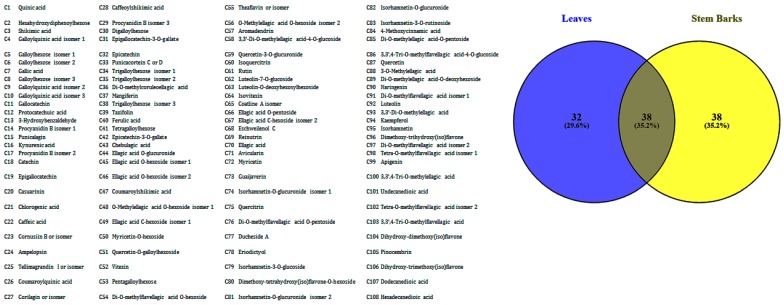
Venn diagram representing the overlap of compounds on the two organs.

An unsupervised multiple correspondence analysis (MCA), a Heatmap clustering approach, and Venn graph were applied on the chemical composition of *A. leiocarpus* samples to obtain a typology of the samples and to characterize the chemical profile differences among those samples. MCA is commonly used qualitative variables to examine a set of observations described by a set of nominal variables. Figure 1A displays the proportion of explained inertia per component and the projection of the samples and chemical compounds on the first two dimensions. Two components were required to summarize approximately 97.7% of the variance. The first component explained 96,1% of variance, while the second accounted for 1, 6%. As we could notice, the samples were predominantly separated by the first component of MCA, with the extracts of stem bark being grouped on the negative side of the factors and the leaves extract on the positive side (Figure 1B). In agreement with MCA, Heatmap separated and categorized the samples into two groups, with each group being divided into two sub-clusters (Figure 1C). On the other hand, we observed that the stem bark extracts were more homogeneous than the leaves extracts. Indeed, ethyl acetate extract of leaves was clearly separated from the other two extracts (methanol and water), which were close (Figure 1B). This indicates that solvent used have a large influence on the leaves secondary metabolite extraction than that of stem bark. Moreover, when cross-checking a list of 108 compounds that were identified in all the leaves extracts against those were recognized in all stem bark extracts, we observed that a total of 38 were commonly detected in the two organs, whereas there were 38 and 32 unique compounds found in stem bark and leaves, respectively (Figure 2).

**Figure 3:**
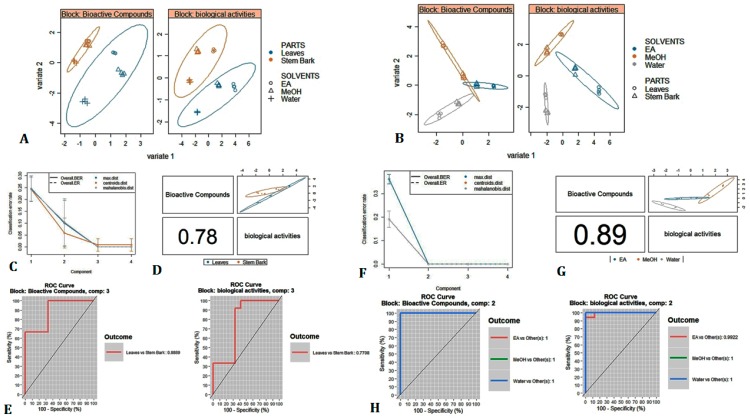
N-integration across multiple datasets analysis on *Anogeissus leiocarpus* bioactive compounds content and biological activities according to two factors (solvents and parts). (**A**,**B**): Sample plot with confidence ellipse according to the parts of plant and the extracting solvent as factor, respectively. (**C**,**F**): The model performance per component for Centroids Distance using 5-fold CV repeated 10 times. (**D**,**G**): the global overview of the relationship between the two datasets at the two first component level. (**E**,**H**): AUC (area under the curve) average and ROC (Receiver Operating Characteristic Curve) curve using one-vs-all comparisons.

The analysis showed the variation of chemical composition of *A. leiocarpus* depending to part used, as well as the influence of solvent on the extraction of compounds, with a more pronounced effect on the leaves. In agreement with this observation, we decided to ascertain whether the plant parts and solvent types used had any statistically significant effect on both total bioactive compounds content and biological activities of *A. leiocarpus.* Thus, multivariate methods for integrative large biological data sets, namely DIABLO, was applied to total bioactive compounds content and biological activities data-sets. DIABLO is a highly flexible supervised multivariate method that enables to classify in an optimal and reliable manner the studied samples and to construct a predictive multi-omics model that can be used to classify new samples. Figure 3 shows the multivariate analysis results. From Figure 3A,B, it is clear that there were parts and solvents effects on the total bioactive compounds content and biological activities of *A. leiocarpus.* In fact, as we could observe in Figure 3A, the factorial plan discriminated leaves parts with the stem bark parts effectively in both bioactive compounds and biological activities data sets. A similar outcome was provided with the second studied factor, in short, a clear segregation between the solvents was achieved (Figure 3B). Furthermore, a better separation of solvents was found while using biological activities data than when using the bioactive compounds data. The different extracts of the stem bark were relatively close as we have seen on the plots. As well by observing the samples plot using bioactive compounds content data (Figure 3A,B, Block: Bioactive compounds), this view was echoed with a consolidation of stem bark extracts contrast to a high variability between the leaves extracts. Accordingly, the extraction solvent, by extension the change of polarity, greatly influenced the bioactive compounds content of leaves than those of stem bark. The present result indicated that *A. leiocarpus* leaves, unlike stem bark, contain chemical molecules with varying polarity and solubility that are sensitive to the variation of solvent.

Figure 3D,G shows that the first three and two components, respectively, of bioactive compounds dataset were positively correlated to biological activities dataset, which allowed for us to say that DIABLO analysis was able to model a good agreement between our datasets. Subsequently, to compare DIABLO models that include/exclude the repeated measures experimental design, we examined the ROC assay (Receiver Operating Characteristic Curve). As we could observe in Figure 3E, the AUC (area under the curve) for the first three component for bioactive compounds and biological activities were 0.89 and 0.77, respectively. As for the second model, the AUC for the first two component were 1 for both bioactive compounds and biological activities (Figure 3H). Finally, the performance of each model was evaluated by estimating the classification error rate. Centroids distance was used as prediction distance and 10 × 5-fold CV as repeated stratified cross-validation. Thus, by observing Figure 3C,F, the best performance was obtained for 3 and 2 component, respectively, which suggests a satisfactory result on our model.

**Figure 4:**
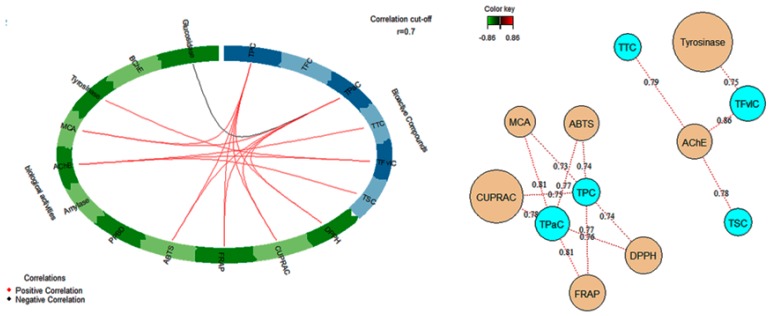
Circos-plot and network showing the relationship between total bioactive compounds and evaluated biological activities (cut-off: *r* = 0.7).

Circos plot and network were carried out to analyze the correlative relationships between total bioactive compound contents of each extract with their biological activities (Figure 4). The analysis revealed that AChE had a positive correlation with total tannin content (TTC) (*r* = 0.79), total saponin content (TSC) (*r* = 0.78) and total flavonol content (TFvLC) (*r* = 0.86), whereas tyrosinase was correlated with TFvLC (*r* = 0.75). Likewise total phenolic content (TPC) and total phenolic acid (TPaC) were positively related to Radical Scavenging Activity ABTS (*r* = 0.74; *r* = 0.77) and DPPH (*r* = 0.74; *r* = 0.77), Reducing Power ability FRAP (*r* = 0.77; *r* = 0.81) and CUPRAC (*r* = 0.75; *r* = 0.78) and Ferrous ion Chelation (MCA) (*r* = 0.73; *r* = 0.81). Accordingly, it was obvious that the phenolic compounds especially phenolic acid compounds were mostly responsible for the antioxidant activities and metal chelating ability of *A. leiocarpus* extracts.

### 3.8. Pharmacological Studies

**Figure 5:**
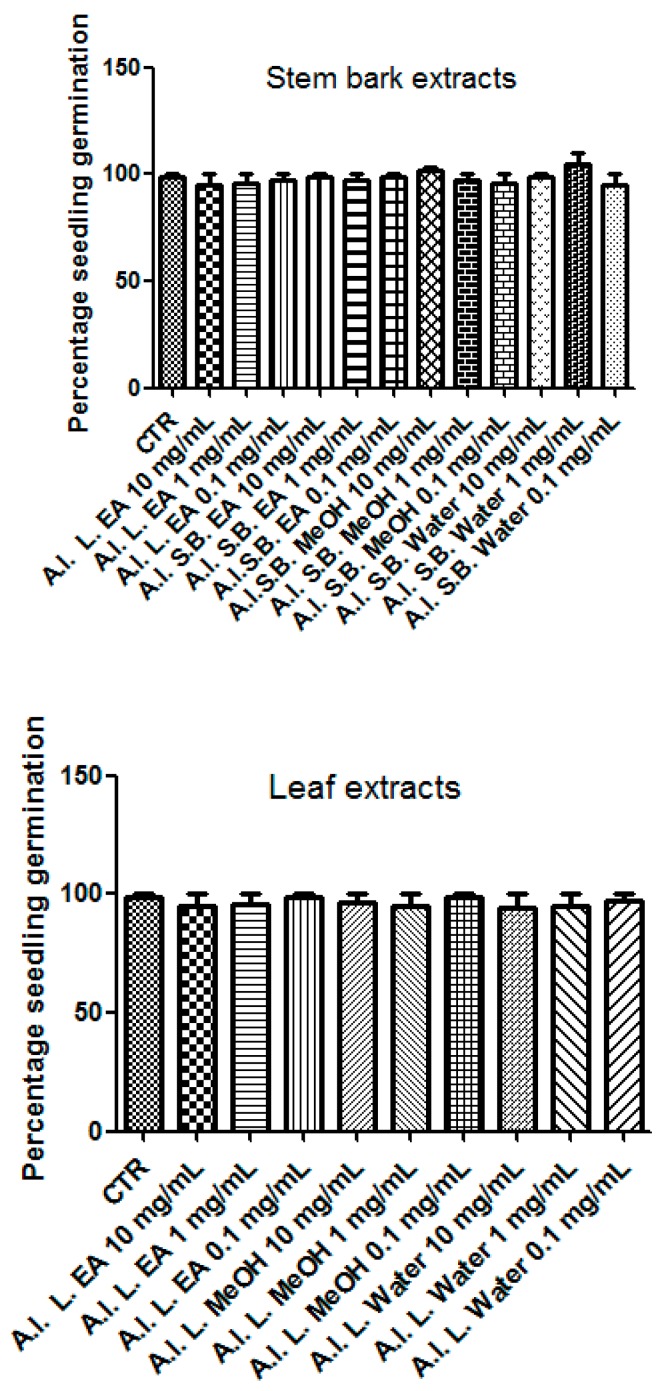
Effects of *A. leiocarpus* leaf (L) and stem bark (SB) extracts (0.1–10 mg/mL) on *Lollo bionda* lettuce root elongation rate. Data, expressed as mean length distribution of germinated seeds, are means ± SD of three experiments performed in triplicate. After exposing lettuce roots to the extracts, a null effect on seedling germination was observed. EA: Ethyl acetate; MeOH: Methanol.

With the aim of investigating extract biological activity, EA, MeOH, and water extracts of *A. leiocarpus* leaves and stem barks were assayed through the allelopathy test. To this regard, the seeds of the commercial *Lollo bionda* lettuce cultivar were exposed to scalar extract concentrations (0.1–10 mg/mL), and the seedling germination and growth were monitored. After incubation of seeds with extracts, we observed a null effect on the seedling germination (Figure 5), thus obtaining a preliminary index of the extract biocompatibility.

**Figure 6:**
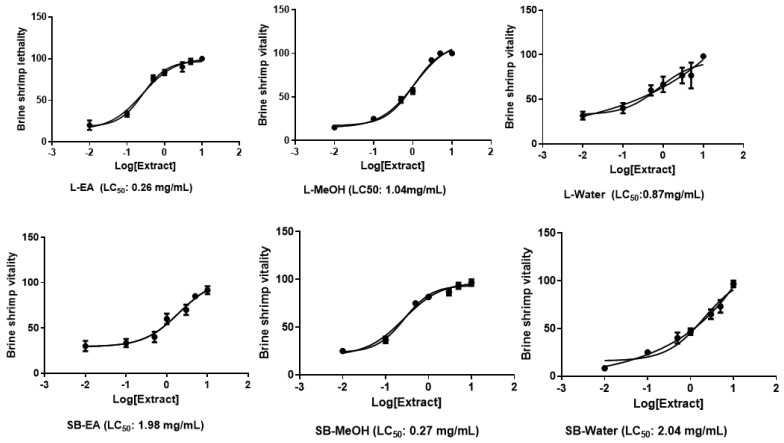
Effects of *A. leiocarpus* leaf (L) and stem bark (SB) extracts (0.01–10 mg/mL) on *Artemia salina* Leach lethality (Brine shrimp lethality test). Data are means ± SD of three experiments performed in triplicate. After exposing brine shrimps to the extracts, LC_50_ values in the range 0.26–2.04 mg/mL were recorded. EA: Ethyl acetate; MeOH: Methanol.

As a further approach to evaluate potential toxicity, *A. leiocarpus* extracts, in the concentration range 0.01–10 mg/mL, were tested on brine shrimp lethality assay, performed on the brine shrimp *Artemia salina* Leach, which is recognized as a valuable tool to predict potential cytotoxicity related to plant extracts [44]. The experimental procedure was conducted in agreement with a previous published paradigm [45]. The results of this assay indicated LC_50_ values in the range 0.26–2.04 mg/mL (Figure 6), which were indicatory to choose the extract concentration for the in vitro and ex vivo investigations in order to elucidate putative protective effects, in the colon.

**Figure 7:**
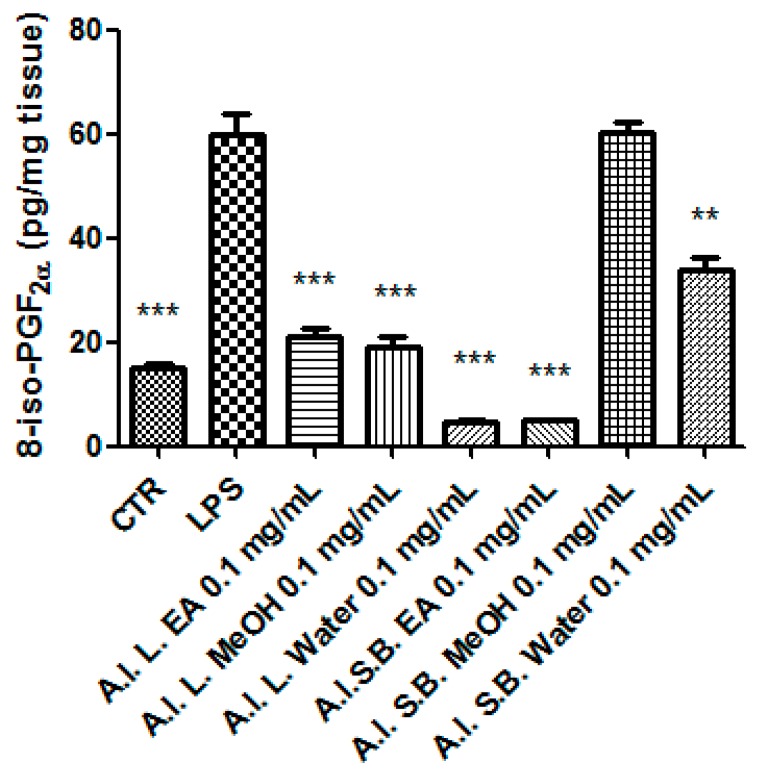
Effect *A. leiocarpus* leaf (L) and stem bark (SB) extracts (0.01 mg/mL) on lipopolysaccharide (LPS)-induced 8-iso-prostaglandin(PG)F_2α_ level in isolated rat colon. EA: Ethyl acetate; MeOH: Methanol. ANOVA, *p* < 0.0001; post hoc, **p < 0.01, ***p < 0.001 vs. LPS.

**Figure 8:**
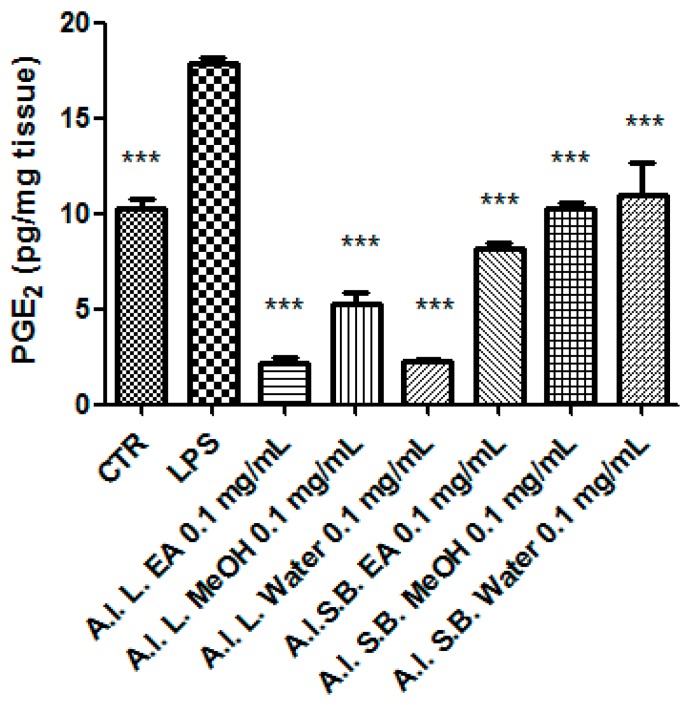
Effect *A. leiocarpus* leaf (L) and stem bark (SB) extracts (0.01 mg/mL) on lipopolysaccharide (LPS)-induced prostaglandin(PG)E_2_ level in isolated rat colon. EA: Ethyl acetate; MeOH: Methanol. ANOVA, *p* < 0.0001; post hoc, ***p < 0.001 vs. LPS.

**Figure 9:**
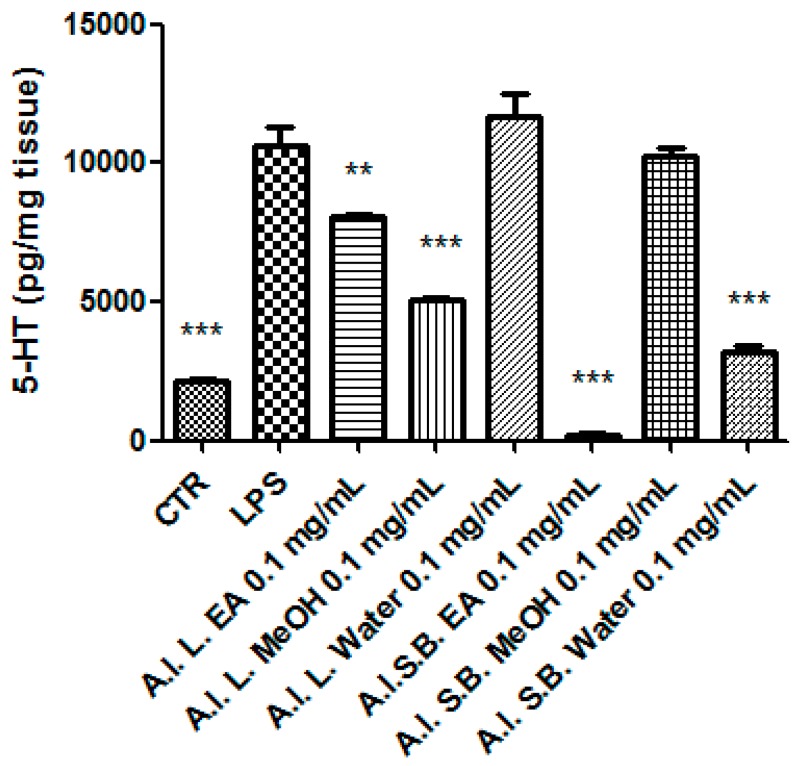
Effect *A. leiocarpus* leaf (L) and stem bark (SB) extracts (0.01 mg/mL) on lipopolysaccharide (LPS)-induced serotonin (5-HT) level in isolated rat colon. EA: Ethyl acetate; MeOH: Methanol. ANOVA, *p* < 0.0001; post hoc, **p < 0.01, ***p < 0.001 vs. LPS.

**Figure 10:**
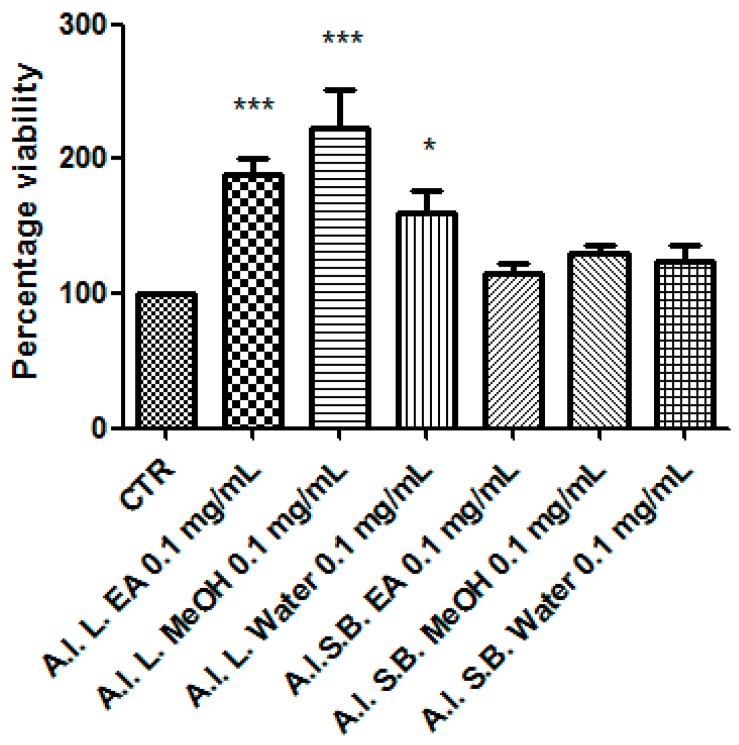
Effect *A. leiocarpus* leaf (L) and stem bark (SB) extracts (0.01 mg/mL) on human colon cancer HCT116 cell line viability (MTT assay). EA: Ethyl acetate; MeOH: Methanol. ANOVA, *p* < 0.0001; post hoc, *p < 0.05, ***p < 0.001 vs. CTR (Control group).

**Figure 11:**
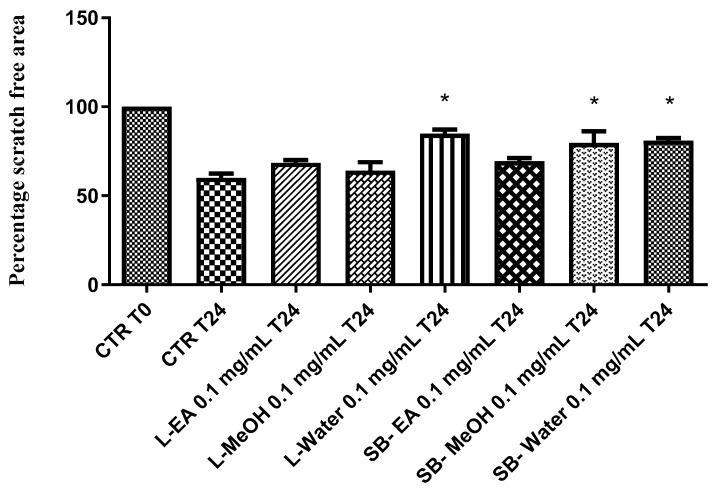
Effect *A. leiocarpus* leaf (L) and stem bark (SB) extracts (0.01 mg/mL) on human colon cancer HCT116 cell line spontaneous migration (wound healing assay). EA: Ethyl acetate; MeOH: Methanol. ANOVA, *p* < 0.01; post hoc, *p < 0.05 vs. CTR T24 (Control group).

Particularly, we selected the concentration 0.1 mg/mL that was at least two-fold lower than LC_50_ and in agreement with previous investigations that demonstrated the antioxidant effects on isolated porcine tissue [46]. While considering these findings, we assayed extract effects on rat colon stimulated with LPS, ex vivo, in order reproduce the burden of oxidative stress and inflammation that characterize ulcerative colitis [20,47,48]. All extracts, with the only exception of stembark methanol extract, revealed effective in reducing LPS-induced 8-iso-PGF_2α_ level (Figure 7). On the other hand, all of the extracts blunted LPS-stimulated PGE_2_ colon level (Figure 8), whereas leaf water and stem bark methanol extracts failed to reduce 5-HT concentration (Figure 9). Finally, when the extracts were tested on colon cancer HCT116 cell line, only stem bark ethyl acetate extract revealed biocompatibility, exerting a null effect on cell proliferation (Figure 10 and Figure 11). Conversely, the other extracts displayed stimulatory effects on either viability or spontaneous migration of HCT116 cells.

## 4. Discussion

Oxidative stress is characterized by the overproduction of reactive oxygen/nitrogen (ROS/RNS) species that could drive to lipid peroxidation [49], which displays a key role in the pathogenesis of ulcerative colitis [50]. 8-iso-PGF_2α_, deriving from ROS/RNS peroxidation of membrane arachidonic acid, represents a stable marker of lipid peroxidation and tissue damage, in vivo [51], whereas the blunting effects on 8-iso-PGF_2α_ production that are induced by herbal extracts were related to protective effects [48,52]. Consistently with the reported antioxidant effects and the findings by Belemnaba et al. [46], the tested extracts blunted LPS-induced 8-iso-PGF_2α_ production, with the only exception being represented by stem bark MeOH extract. On the other hand, stem bark MeOH extract showed the ability to reduce the level of malondialdehyde (MDA), which is another key marker of lipid peroxidation, in vivo [53]. This discrepancy could depend on more than one speculation. On one side, the differences in the employed experimental ex vivo and in vivo paradigms that were chosen by us and Akanbi and colleagues [53], respectively, could be crucial. On the other side, we should also consider that isoprostanes derive only by arachidonic acid peroxidation, whereas MDA could originate from various polyunsaturated acids [54], and this could represent a limit in the evaluation of lipid peroxidation.

The effects of *A. leiocarpus* extracts on LPS-induced levels of colon PGE_2_ were investigated as well. PGE_2_ is a cyclooxygenase (COX)-2-derived pro-inflammatory mediator, whose upregulation has been long involved in colon inflammation and damage, whereas the antioxidants were revealed to be effective in blunting the colon levels of this prostaglandin [47,55]. All of the tested extracts proved able in down-regulating LPS-induced PGE_2_ level (Figure 8), consistently with the reported antioxidant effects.

A blunting effect on LPS-induced 5-HT level was observed after treating the colon specimens with *A. leiocarpus* extract, as well. 5-HT pro-inflammatory role in ulcerative colitis was previously suggested [56], which possibly involved the activation of 5-HT_3_ receptors [57]. With the only exception of leaf water and stem bark MeOH extracts, the *A. leiocarpus* extracts displayed a significant inhibition of 5-HT steady state level, in the colon. This could be, albeit partially, related to decreased neurotransmitter synthesis and release, in the colon tissue [58,59].

Collectively, all of the tested *A. leiocarpus* extracts could play a noteworthy anti-inflammatory role, as indicated by their blunting effects on LPS-stimulated PGE_2_ level. On the other hand, the lack of efficacy that was exerted by the leaf water and stem bark MeOH extracts on LPS-induced levels of 5-HT and 8-iso-PGF_2α_ suggests that the lower quantitative profile of gallic acid, catechin, and epicatechin could limit the antioxidant potency, as compared to the other tested extracts.

Finally, the extracts were tested for their putative anti-proliferative role against the human colon HCT116 cell line, which was previously found to be sensitive to different polarity extracts from *A. latifolia* [12]. In the present study, the anti-proliferative effects were investigated through validated in vitro tests, including MTT and wound healing assays. A different pattern of effects on cell viability was observed after exposing HCT116 cells to *A. leiocarpus* extracts. On one side, stem bark extracts displayed a null effect on cell viability (Figure 10), which resulted in the range of biocompatibility (>70% and <130% as compared to control group). On the other side, leaf extract increased significantly HCT116 cell viability (>140% as compared to control group). Actually, the stimulating effect on HCT116 cell viability induced by leaf extracts could be related to the their higher qualitative content of metabolites that are related to quercetin, which was found to exert protective effects on this cell line [60]. Additionally, water leaf and water and MeOH stem bark extracts induced spontaneous HCT116 cell migration in wound healing assay (Figure 11), thus also indicating a potential stimulating-effect on invasion capacity. Conversely, stem bark EA and leaf EA and MeOH extracts did not exert any influence on spontaneous HCT116 cell migration, in the 24 h following experimental lesion induced on cell monolayer. Actually, the different effects that were showed by the tested extracts in the wound healing paradigm could be related, at least in part, to the different content of gallic acid, which was found to inhibit spontaneous cell migration [61]. As a conclusive note of the pharmacological investigation, it resulted of particular interest the antioxidant/anti-inflammatory profile that was exerted by the stem bark EA extract, together with its null effect on colon cancer cell proliferation. The highest inhibitory effect that is exerted by this extract on colon 5-HT level could be one of the main causes leading to the null effect on HCT116 viability and spontaneous migration [62].

## 5. Conclusions

Data that are presented in this study highlighted the key role of solvent choice in the quest for novel bioactive compounds from plants. It was demonstrated that water and methanol were good solvents for the extraction of phytochemicals having antioxidant properties. The methanol extract of *A. leiocarpus* leaves was also an active cholinesterase inhibitor, while the methanol extract of the stem bark inhibited tyrosinase. On the other hand, the ethyl acetate extract of *A. leiocarpus* leaves and stem bark showed potent inhibition against α-glucosidase. The pharmacological study that was carried out on isolated colon and HCT116 cell line further deepened the spectrum of potential application of the present extracts. Noteworthy interest derives from the stem bark EA extract that, besides exerting the best antioxidant/anti-inflammatory profile, was the only one that was unable to stimulate the proliferation of human colon cancer HCT116 cell line, thus supporting potential application in the prevention of the oxidative stress-induced tissue damage occurring in ulcerative colitis. As a conclusion, *A. leiocarpus* stem bark EA extract represents a potential source of bioactive compounds for the development of novel therapeutic agents.

## Figures and Tables

**Table 1:** Quantitative phytochemical determinations of *A. leiocarpus* leaves and stem bark extracts.

Samples	Total Phenolic Content (mg GAE/g)	Total Flavonoid Content (mg RE/g)	Total Flavonol (mg CE/g)	Total Phenolic acid (mg CAE/g)	Total Tannin Content (mg CE/g)	Total Saponin Content (mgQE/g)
Leaves-EA	49 ± 1^e^	35.0 ± 0.6^c^	6.0 ± 0.1^d^	nd	6.0 ± 0.4^e^	190 ± 17^bc^
Leaves-MeOH	223 ± 2^c^	54.0 ± 0.6^b^	79 ± 3^a^	8 ± 1^b^	77.0 ± 0.7^a^	438 ± 54^a^
Leaves-Water	257 ± 3^b^	89.0 ± 0.2^a^	3.46 ± 0.02^e^	14 ± 1^a^	18 ± 4^c^	200 ± 30^bc^
Stem barks-EA	207 ± 2^d^	16 ± 0.3^f^	14.0 ± 0.3^c^	nd	17 ± 0.5^c^	171 ± 24^bc^
Stem barks-MeOH	271 ± 1^a^	27.0 ± 0.3^e^	28.0 ± 0.4^b^	nd	33 ± 1^b^	230 ± 34^b^
Stem barks-Water	274 ± 2^a^	33.0 ± 0.2^d^	2.09 ± 0.01^e^	7.0 ± 0.6^c^	10.0 ± 0.1^d^	163 ± 28^c^

Values expressed are means ± SD of three parallel measurements. GAE: Gallic acid equivalent; RE: Rutin equivalent. CE: Catechin equivalent; CAE: Caffeic acid equivalent; QE: Quillaja equivalent. nd: not detected. Superscripts in the same column indicate significant difference in the tested extracts (*p* < 0.05).

**Table 2:** Phenol content of *A. leiocarpus* leaves and stem bark ethyl acetate (EA), methanol (MeOH) and water extracts.

Phenolic Compound	Leaves-EA mg/g	Leaves-MeOH mg/g	Leaves-Water Extract mg/g	Stem Barks-EA mg/g	Stem Barks MeOH mg/g	Stem Barks-Water mg/g
Gallic acid	226 ± 21	89 ± 28	30 ± 3	65 ± 6	38 ± 2	37 ± 1
Catechin	7.0 ± 0.8	3.0 ± 0.2	9 ± 1	1.0 ± 0.1	0.29 ± 0.02	0.28 ± 0.02
Epicatechin	0.29 ± 0.02	2.0 ± 0.1	0.27 ± 0.02	0.27 ± 0.02	0.18 ± 0.01	1.0 ± 0.1

**Table 3:** Chemical composition of *A. leiocarpus* extracts.

No.	Name	Formula	[M + H]^+^	[M – H]^–^	Leaves-EA	Leaves-MeOH	Leaves-Water	Stem Bark-EA	Stem Bark-MeOH	Stem Bark-Water
1	Quinic acid	C_7_H_12_O_6_		19,105,557	+	+	+	+	+	+
2	Hexahydroxydiphenoylhexose	C_20_H_18_O_14_		48,106,184	–	–	–	+	+	+
3	Shikimic acid	C_7_H_10_O_5_		17,304,500	+	+	+	+	+	+
4	Galloylquinic acid isomer 1	C_14_H_16_O_10_		34,306,653	–	–	–	–	+	+
5	Galloylhexose isomer 1	C_13_H_16_O_10_		33,106,653	–	–	–	+	+	+
6	Galloylhexose isomer 2	C_13_H_16_O_10_		33,106,653	–	–	–	+	+	+
7^1^	Gallic acid (3,4,5-Trihydroxybenzoic acid)	C_7_H_6_O_5_		16,901,370	+	+	+	+	+	+
8	Galloylhexose isomer 3	C_13_H_16_O_10_		33,106,653	–	–	–	+	+	+
9	Galloylquinic acid isomer 2	C_14_H_16_O_10_		34,306,653	–	–	–	–	+	+
10	Galloylquinic acid isomer 3	C_14_H_16_O_10_		34,306,653	–	–	–	–	+	+
11	Gallocatechin	C_15_H1_4_O_7_		30,506,613	–	+	+	+	+	+
12	Protocatechuic acid (3,4-Dihydroxybenzoic acid)	C_7_H_6_O_4_		15,301,879	+	+	+	+	+	+
13	3-Hydroxybenzaldehyde	C_7_H_6_O_2_	12,304,461		+	+	+	–	–	–
14	Procyanidin B isomer 1	C_30_H_26_O_12_		57,713,460	–	–	–	+	+	+
15	Punicalagin	C_48_H_28_O_30_		108,305,872	+	+	+	+	+	+
16	Kynurenic acid	C_10_H_7_NO_3_	19,005,042		–	+	+	–	–	–
17	Procyanidin B isomer 2	C_30_H_26_O_12_		57,713,460	–	–	–	+	+	+
18^1^	Catechin	C_15_H_14_O_6_		28,907,121	+	+	+	+	+	+
19^1^	Epigallocatechin	C_15_H_14_O_7_		30,506,613	–	+	+	+	+	+
20	Casuarinin	C_41_H_28_O_26_		93,507,906	+	+	+	+	+	+
21	Chlorogenic acid (3-O-Caffeoylquinic acid)	C_16_H_18_O_9_	35,510,291		+	+	+	–	–	–
22	Caffeic acid	C_9_H_8_O_4_		17,903,444	+	+	+	+	+	+
23	Cornusiin B or isomer	C_48_H_30_O_30_		108,507,437	+	+	+	+	+	–
24	Ampelopsin (Dihydromyricetin)	C_15_H_12_O_8_		31,904,540	+	+	+	+	+	+
25	Tellimagrandin I or isomer	C_34_H_26_O_22_		78,508,375	+	+	+	+	+	+
26	Coumaroylquinic acid	C_16_H_18_O_8_		33,709,235	+	+	+	–	–	–
27	Corilagin or isomer	C_27_H_22_O_18_		63,307,279	+	+	+	–	–	–
28	Caffeoylshikimic acid	C_16_H_16_O_8_		33,507,670	–	+	+	–	–	–
29	Procyanidin B isomer 3	C_30_H_26_O_12_		57,713,460	–	–	–	+	+	–
30	Digalloylhexose	C_20_H_20_O_14_		48,307,749	–	–	–	+	+	+
31^1^	Epigallocatechin-3-O-gallate (Teatannin II)	C_22_H_18_O_11_		45,707,709	–	+	+	+	+	+
32^1^	Epicatechin	C_15_H_14_O_6_		28,907,121	–	+	+	+	+	+
33	Punicacortein C or D	C_48_H_28_O_30_		108,305,872	–	–	–	+	+	+
34	Trigalloylhexose isomer 1	C_27_H_24_O_18_		63,508,844	–	–	–	+	+	+
35	Trigalloylhexose isomer 2	C_27_H_24_O_18_		63,508,844	–	–	–	+	+	+
36	Di-O-methylcoruleoellagic acid	C_16_H_10_O_10_		36,101,958	–	–	–	+	–	–
37	Mangiferin	C_19_H_18_O_11_		42,107,709	–	–	–	+	+	+
38	Trigalloylhexose isomer 3	C_27_H_24_O_18_		63,508,844	–	–	–	+	+	+
39^1^	Taxifolin (Dihydroquercetin)	C_15_H_12_O_7_		30,305,048	+	+	+	+	+	+
40	Ferulic acid	C_10_H_10_O_4_		19,305,009	+	+	+	–	–	–
41	Tetragalloylhexose	C_34_H_28_O_22_		78,709,940	–	–	–	+	+	–
42^1^	Epicatechin-3-O-gallate	C_22_H_18_O_10_		44,108,218	+	+	+	+	+	+
43	Chebulagic acid	C_41_H_30_O_27_		95,308,963	+	+	+	–	–	–
44	Ellagic acid O-glucuronide	C_20_H_14_O_14_		47,703,054	–	–	–	+	+	+
45	Ellagic acid O-hexoside isomer 1	C_20_H_16_O_13_		46,305,127	+	+	+	+	+	+
46	Ellagic acid O-hexoside isomer 2	C_20_H_16_O_13_		46,305,127	+	+	+	+	+	+
47	Coumaroylshikimic acid	C_16_H_16_O_7_		31,908,178	+	+	+	–	–	–
48	O–Methylellagic acid O-hexoside isomer 1	C_21_H_18_O_13_		47,706,692	–	–	–	+	+	+
49	Ellagic acid C-hexoside isomer 1	C_20_H_16_O_13_	46,506,692		–	–	–	+	+	+
50	Myricetin-O-hexoside	C_21_H_20_O_13_		47,908,257	+	+	+	+	+	+
51	Quercetin-O-galloylhexoside	C_28_H_24_O_16_		61,509,862	+	+	+	–	–	–
52	Vitexin (Apigenin-8-C-glucoside)	C_21_H_20_O_10_	43,311,348		–	–	–	+	+	+
53	Pentagalloylhexose	C_41_H_32_O_26_		93,911,036	–	–	–	+	+	–
54	Di-O-methylflavellagic acid O-hexoside	C_22_H_20_O_14_		50,707,749	–	–	–	+	+	+
55	Theaflavin or isomer	C_29_H_24_O_12_	56,513,461		–	+	–	–	+	–
56	O-Methylellagic acid O-hexoside isomer 2	C_21_H_18_O_13_		47,706,692	–	–	–	+	+	+
57	Aromadendrin (Dihydrokaempferol)	C_15_H_12_O_6_		28,705,557	+	+	+	+	+	–
58	3,3′-Di-O-methylellagic acid-4-O-glucoside	C_22_H_20_O_13_		49,108,257	+	+	+	+	+	+
59	Quercetin-3-O-glucuronide	C_21_H_18_O_13_		47,706,692	+	+	+	–	–	–
60	Isoquercitrin (Hirsutrin, Quercetin-3-O-glucoside)	C_21_H_20_O_12_		46,308,765	+	+	+	–	–	–
61	Rutin (Quercetin-3-O-rutinoside)	C_27_H_30_O_16_	61,116,122		+	+	+	–	–	–
62	Luteolin-7-O-glucoside (Cynaroside)	C_21_H_20_O_11_		44,709,274	–	–	–	–	–	+
63	Luteolin-O-deoxyhexosylhexoside	C_27_H_30_O_15_		59,315,065	–	–	–	–	–	+
64	Isovitexin (Apigenin-6-C-glucoside)	C_21_H_20_O_10_	43,311,348		–	–	–	+	+	+
65	Coatline A isomer	C_21_H_24_O_10_		43,512,913	–	+	+	+	+	–
66	Ellagic acid O-pentoside	C_19_H_14_O_12_		43,304,071	–	–	–	+	+	+
67	Ellagic acid C-hexoside isomer 2	C_20_H_16_O_13_	46,506,692		–	–	–	+	+	+
68	Eschweilenol C (Ellagic acid-4-O-rhamnoside)	C_20_H_16_O_12_		44,705,636	+	+	+	+	+	+
69	Reinutrin (Quercetin-3-O-xyloside)	C_20_H_18_O_11_		43,307,709	+	+	+	–	–	–
70	Ellagic acid	C_14_H_6_O_8_		30,099,845	+	+	+	+	+	+
71	Avicularin (Quercetin-3-O-arabinoside)	C_20_H_18_O_11_		43,307,709	+	+	+	–	–	–
72^1^	Myricetin (3,3′,4′,5,5′,7-Hexahydroxyflavone)	C_15_H_10_O_8_		31,702,974	+	+	+	+	+	+
73	Guaijaverin (Quercetin-3-O-arabinoside)	C_20_H_18_O_11_		43,307,709	+	+	+	–	–	–
74	Isorhamnetin-O-glucuronide isomer 1	C_22_H_20_O_13_		49,108,257	–	+	–	–	–	–
75	Quercitrin (Quercetin-3-O-rhamnoside)	C_21_H_20_O_11_		44,709,274	+	+	+	–	–	–
76	Di-O-methylflavellagic acid O-pentoside	C_21_H_18_O_13_		47,706,692	–	–	–	+	–	–
77	Ducheside A (3-O-Methylellagic acid-4′-O-xyloside)	C_20_H_16_O_12_		44,705,636	–	–	–	+	+	+
78	Eriodictyol	C_15_H_12_O_6_		28,705,557	+	+	–	+	+	+
79	Isorhamnetin-3-O-glucoside	C_22_H_22_O_12_		47,710,330	+	+	+	–	–	–
80	Dimethoxy-tetrahydroxy(iso)flavone-O-hexoside	C_23_H_24_O_13_		50,711,387	–	+	+	–	–	–
81	Isorhamnetin-O-glucuronide isomer 2	C_22_H_20_O_13_		49,108,257	–	+	–	–	–	–
82	Isorhamnetin-O-glucuronide	C_22_H_20_O_13_		49,108,257	–	–	+	–	–	–
83	Isorhamnetin-3-O-rutinoside (Narcissin)	C_28_H_32_O_16_		62,316,122	+	+	+	–	–	–
84	4-Methoxycinnamic acid	C_10_H_10_O_3_		17,907,082	–	–	–	–	+	–
85	Di-O-methylellagic acid-O-pentoside	C_21_H_18_O_12_		46,107,200	+	+	+	+	+	+
86	3,3′,4-Tri-O-methylflavellagic acid-4-O-glucoside	C_23_H_22_O_14_		52,109,314	+	+	+	+	+	+
87	Quercetin	C_15_H_10_O_7_		30,103,483	+	+	+	–	–	–
88	3-O-Methylellagic acid	C_15_H_8_O_8_		31,501,410	–	+	+	+	+	+
89	Di-O-methylellagic acid-O-deoxyhexoside	C_22_H_20_O_12_		47,508,766	–	–	–	+	+	+
90^1^	Naringenin	C_15_H_12_O_5_		27,106,065	+	+	+	+	+	+
91	Di-O-methylflavellagic acid isomer 1	C_16_H_10_O_9_		34,502,466	–	–	–	+	+	+
92^1^	Luteolin (3′,4′,5,7-Tetrahydroxyflavone)	C_15_H_10_O_6_		28,503,991	+	+	+	–	+	–
93	3,3′-Di-O-methylellagic acid	C_16_H_10_O_8_		32,902,975	+	+	+	+	+	+
94	Kaempferol (3,4′,5,7-Tetrahydroxyflavone)	C_15_H_10_O_6_	28,705,556		+	+	+	–	–	–
95	Isorhamnetin (3′-Methoxy-3,4′,5,7-tetrahydroxyflavone)	C_16_H_12_O_7_		31,505,048	+	+	+	–	–	–
96	Dimethoxy-trihydroxy(iso)flavone	C_17_H_14_O_7_		32,906,613	+	+	–	–	–	–
97	Di-O-methylflavellagic acid isomer 2	C_16_H_10_O_9_		34,502,466	–	–	–	+	+	+
98	Tetra-O-methylflavellagic acid isomer 1	C_18_H_14_O_9_		37,305,596	–	–	–	+	+	+
99	Apigenin (4′,5,7-Trihydroxyflavone)	C_15_H_10_O_5_		26,904,500	–	+	–	–	–	+
100	3,3′,4-Tri-O-methylellagic acid	C_17_H_12_O_8_		34,304,540	+	+	+	+	+	+
101	Undecanedioic acid	C_11_H_20_O_4_		21,512,834	+	+	+	–	–	–
102	Tetra-O-methylflavellagic acid isomer 2	C_18_H_14_O_9_		37,305,596	–	–	–	+	+	+
103	3,3′,4-Tri-O-methylflavellagic acid	C_17_H_12_O_9_		35,904,031	+	+	+	+	+	+
104	Dihydroxy-dimethoxy(iso)flavone	C_17_H_14_O_6_		31,307,122	+	+	–	–	–	–
105	Pinocembrin (5,7-Dihydroxyflavanone)	C_15_H_12_O_4_		25,506,573	+	+	–	–	+	–
106	Dihydroxy-trimethoxy(iso)flavone	C_18_H_16_O_7_		34,308,178	+	+	+	–	–	–
107	Dodecanedioic acid	C_12_H_22_O_4_		22,914,399	+	+	+	–	–	–
108	Hexadecanedioic acid	C_16_H_30_O_4_		28,520,659	+	+	–	–	–	–

^1^ Confirmed by standard.

**Table 4:** Antioxidant properties of *A. leiocarpus* extracts.

Samples	DPPH (mmol TE/g)	ABTS (mmol TE/g)	CUPRAC (mmol TE/g)	FRAP (mmol TE/g)	Metal Chelating (mg EDTAE/g)	Phosphomolybdenum (mmol TE/g)
Leaves-EA	30 ± 0.01^f^	0.26 ± 0.02^e^	0.50 ± 0.04^e^	0.26 ± 0.03^d^	10.0 ± 0.8^f^	2.0 ± 0.1^e^
Leaves-MeOH	5.0 ± 0.1^d^	3.0 ± 0.4^c^	7.0 ± 0.2^c^	4.0 ± 0.1^c^	47.0 ± 0.8^c^	4.0 ± 0.1^d^
Leaves-Water	5.0 ± 0.1^c^	4.0 ± 0.3^b^	7.0 ± 0.2^b^	6.0 ± 0.4^b^	79.0 ± 0.9^a^	4.0 ± 0.1^d^
Stem barks-EA	3.0 ± 0.1^e^	2.0 ± 0.1^d^	5.0 ± 0.2^d^	4.0 ± 0.1^c^	30.0 ± 0.5^e^	4.0 ± 0.1^c^
Stem barks-MeOH	6.0 ± 0.1^a^	5.0 ± 0.1^a^	8.0 ± 0.2^a^	6.0 ± 0.3^a^	45.0 ± 0.6^d^	6.0 ± 0.2^a^
Stem barks-Water	5.0 ± 0.1^b^	4.0 ± 0.4^b^	8.0 ± 0.1^b^	6.0 ± 0.2^a^	61.0 ± 0.4^b^	5.0 ± 0.1^a^

Values expressed are means ± S.D. of three parallel measurements. DPPH: 2-diphenyl-1-picrylhydrazyl; ABTS: 2,2’-azino-bis(3-ethylbenzothiazoline-6-sulphonic acid; CUPRAC: cupric reducing antioxidant capacity; FRAP: ferric reducing antioxidant power; TE: Trolox equivalent; EDTAE: ethylenediaminetetraacetic acid equivalent. Superscripts in the same column indicate significant difference in the tested extracts (*p* < 0.05).

**Table 5:** Enzyme inhibitory properties of *A. leiocarpus* extracts.

Samples	AChE Inhibition (mg GALAE/g)	BChE Inhibition (mg GALAE/g)	Tyrosinase Inhibition (mg KAE/g)	Amylase Inhibition (mmol ACAE/g)	Glucosidase Inhibition (mmol ACAE/g)
Leaves-EA	4.0 ± 0.2^d^	3.0 ± 0.2^b^	131.0 ± 0.2^d^	0.79 ± 0.04^b^	15.0±0.1^b^
Leaves-MeOH	4.68 ± 0.02^a^	4.0 ± 0.1^a^	154.0 ± 0.2^b^	1.0 ± 0.1^a^	nd
Leaves-Water	4.19 ± 0.04^c^	nd	113 ± 1^e^	0.53 ± 0.04^c^	nd
Stem barks-EA	4.0 ± 0.1^bc^	2.0 ± 0.2^c^	152.0 ± 0.6^c^	1.0 ± 0.1^b^	15.0 ± 0.1^a^
Stem barks-MeOH	4.0 ± 0.1^b^	1.0 ± 0.3^c^	155.26 ± 0.04^a^	0.85 ± 0.03^b^	nd
Stem barks-Water	4.0 ± 0.1^e^	0.5 ± 0.1^d^	113.0 ± 0.6^e^	0.19 ± 0.01^d^	nd

Values expressed are means ± S.D. of three parallel measurements. AChE: Acetylcholinesterase; BChE: Butyrylcholinesterase; GALAE: Galatamine equivalent; KAE: Kojic acid equivalent; ACAE: Acarbose equivalent. nd: not detected. Superscripts in the same column indicate significant difference in the tested extracts (*p* <0.05).

## Supplementary Materials

The following are available online at https://www.mdpi.com/2076-3921/8/9/343/s1. Table S1: Chemical composition of *A. leiocarpus* leaves ethyl acetate extract. Table S2: Chemical composition of *A. leiocarpus* leaves methanol extract. Table S3: Chemical composition of *A. leiocarpus* leaves water extract. Table S4: Chemical composition of *A. leiocarpus* stem bark ethyl acetate extract. Table S5: Chemical composition of *A. leiocarpus* stem bark methanol extract. Table S6: Chemical composition of *A. leiocarpus* stem bark water extract.
