# Supplementary material for: Qualitative Chemical Characterization and Multidirectional Biological Investigation of Leaves and Bark Extracts of Anogeissus leiocarpus (DC.) Guill. & Perr. (Combretaceae)
Source: Antioxidants (Basel). 2019 Sep 1;8(9):343. doi: 10.3390/antiox8090343 (PMC6770311; doi:10.3390/antiox8090343)
Supplement: Supplementary file 1 [file antioxidants-08-00343-s001.pdf]

Table S1. Chemical composition of *A. leiocarpus* leaves ethyl acetate extract.

No.	Name	Formula	Rt	$[M + H]^+$	$[M – H]^–$	Fragment 1	Fragment 2	Fragment 3	Fragment 4	Fragment 5	Literature
1	Quinic acid	$C_7H_{12}O_6$	122		19,105,557	1,730,445	1,270,388	1,110,439	930,331	850,280	
2	Shikimic acid	$C_7H_{10}O_5$	126		17,304,500	1,550,339	1,370,234	1,110,437	930,331	730,280	
31	Gallic acid (345-Trihydroxybenzoic acid)	$C_7H_6O_5$	231		16,901,370	1,250,231	970,279	810,331	790,175	690,330	[1]
4	Protocatechuic acid (34-Dihydroxybenzoic acid)	$C_7H_6O_4$	466		15,301,879	1,090,281	1,080,202	910,176	810,332		[1]
5	3-Hydroxybenzaldehyde	$C_7H_6O_2$	1193	12,304,461		1,050,451	950,497	810,342			
6	Punicalagin	$C_{48}H_{28}O_{30}$	1201		108,305,872	7,810,529	7,210,309	6,009,910	5,750,098	3,009,995	[2]
71	Catechin	$C_{15}H_{14}O_6$	1326		28,907,121	2,450,823	2,050,500	2,030,699	1,250,231	1,090,278	
8	Casuarinin	$C_{41}H_{28}O_{26}$	1363		93,507,906	9,170,717	7,830,693	6,330,734	3,009,998	2,750,201	
91	Chlorogenic acid (3-O-Caffeoylquinic acid)	$C_{16}H_{18}O_9$	1418	35,510,291		1,630,392	1,450,285	1,350,444	1,170,338	890,391	
10	Caffeic acid	$C_9H_8O_4$	1428		17,903,444	1,350,440	1,070,489				
11	Cornusiin B or isomer	$C_{48}H_{30}O_{30}$	1450		108,507,437	9,330,720	7,830,693	6,310,602	4,509,947	3,009,997	
12	Ampelopsin (Dihydromyricetin)	$C_{15}H_{12}O_8$	1463		31,904,540	3,010,361	1,930,136	1,789,975	1,530,182	1,250,230	
13	Tellimagrandin I or isomer	$C_{34}H_{26}O_{22}$	1506		78,508,375	6,330,751	6,150,630	4,830,761	3,009,994	2,750,194	
14	Coumaroylquinic acid	$C_{16}H_{18}O_8$	1681		33,709,235	1,910,556	1,730,447	1,630,387	1,190,486	930,331	
15	Corilagin or isomer	$C_{27}H_{22}O_{18}$	1747		63,307,279	4,630,502	4,190,630	3,009,994	2,750,199	1,690,132	
161	Taxifolin (Dihydroquercetin)	$C_{15}H_{12}O_7$	1922		30,305,048	2,850,410	1,990,395	1,750,391	1,530,181	1,250,231	
17	Ferulic acid	$C_{10}H_{10}O_4$	1924		19,305,009	1,780,260	1,490,596	1,370,233	1,340,362	1,210,279	
181	Epicatechin-3-O-gallate	$C_{22}H_{18}O_{10}$	1934		44,108,218	2,890,714	2,710,612	2,450,813	1,690,132	1,250,234	
19	Chebulagic acid	$C_{41}H_{30}O_{27}$	1961		95,308,963	9,350,833	7,830,655	4,630,522	3,009,993	2,750,209	[1]
20	Ellagic acid O-hexoside isomer 1	$C_{20}H_{16}O_{13}$	1988		46,305,127	3,009,995	2,999,911				
21	Ellagic acid O-hexoside isomer 2	$C_{20}H_{16}O_{13}$	2022		46,305,127	3,009,993	2,999,912				
22	Coumaroylshikimic acid	$C_{16}H_{16}O_7$	2031		31,908,178	1,730,442	1,630,390	1,550,338	1,190,488	1,110,437	
23	Myricetin-O-hexoside	$C_{21}H_{20}O_{13}$	2086		47,908,257	3,170,302	3,160,227	2,870,203	2,710,254	2,420,215	
24	Quercetin-O-galloylhexoside	$C_{28}H_{24}O_{16}$	2154		61,509,862	4,630,888	3,130,574	3,010,357	3,000,279	2,710,249	
25	Aromadendrin (Dihydrokaempferol)	$C_{15}H_{12}O_6$	2180		28,705,557	2,590,606	2,430,659	2010558	1,770,549	1,250,231	
26	33'-Di-O-methylellagic acid-4-O-glucoside	$C_{22}H_{20}O_{13}$	2214		49,108,257	4,760,616	3,280,229	3,129,992	2,979,775		
27	Quercetin-3-O-glucuronide	$C_{21}H_{18}O_{13}$	2266		47,706,692	3,010,357	2,550,300	1,789,977	1,510,025		

281	Isoquercitrin (Hirsutrin Quercetin-3-O-glucoside)	$C21H20O_{12}$	2287		46,308,765	3,010,356	3,000,278	2,710,252	1,789,978	1,510,025	
291	Rutin (Quercetin-3-O-rutinoside)	$C_{27}H_{30}O_{16}$	2295	61,116,122		4,651,026	3,030,503	1,290,548	850,291		
30	Eschweilenol C (Ellagic acid-4-O-rhamnoside)	$C_{20}H_{16}O_{12}$	2306		44,705,636	3,009,994	2,999,916				[2]
31	Reinutrin (Quercetin-3-O-xyloside)	$C_{20}H_{18}O_{11}$	2317		43,307,709	3,010,357	3,000,267	2,710,252	1,789,971	1,510,031	
32	Ellagic acid	$C_{14}H6O_{8}$	2335		30,099,845	2,839,966	2,570,089	2,450,095	2,290,140	1,850,239	[2]
33	Avicularin (Quercetin-3-O-arabinoside)	$C_{20}H_{18}O_{11}$	2344		43,307,709	3,010,361	3,000,279	2,710,252	1,789,981	1,510,027	
341	Myricetin (33'4'55'7-Hexahydroxyflavone)	$C_{15}H_{10}O_{8}$	2412		31,702,974	1,920,050	1,789,977	1,650,188	1,510,026	1,370,233	
35	Guaijaverin (Quercetin-3-O-arabinoside)	$C_{20}H_{18}O_{11}$	2419		43,307,709	3,010,356	3,000,277	2,710,242	1,789,972	1,510,021	
361	Quercitrin (Quercetin-3-O-rhamnoside)	$C_{21}H20O_{11}$	2442		44,709,274	3,010,356	3,000,279	2,710,252	1,789,978	1,510,026	
37	Eriodictyol	$C_{15}H_{12}O_{6}$	2482		28,705,557	1,510,026	1,350,441	1,070,125			
381	Isorhamnetin-3-O-glucoside	$C_{22}H_{22}O_{12}$	2488		47,710,330	3,150,514	3,140,437	2,850,409	2,710,252	2,570,456	
39	Isorhamnetin-3-O-rutinoside (Narcissin)	$C_{28}H_{32}O_{16}$	2516		62,316,122	3,150,514	3,140,440	3,000,273	2,990,203	2,710,253	
40	Di-O-methylellagic acid-O-pentoside	$C_{21}H_{18}O_{12}$	2530		46,107,200	3,280,228	3,129,995	2,979,756	2,850,050		
41	33'4-Tri-O-methylflavellagic acid-4-O-glucoside	$C_{23}H_{22}O_{14}$	2553		52,109,314	5,060,715	4,910,469	3,580,333	3,430,098	3,279,863	[3]
421	Quercetin	$C_{15}H_{10}O_{7}$	2692		30,103,483	2,730,407	2450461	1,789,978	1,510,026	1,210,281	
431	Naringenin	$C_{15}H_{12}O_{5}$	2715		27,106,065	2,270,712	1,770,186	1,510,026	1,190,489	1,070,126	
441	Luteolin (3'4'57-Tetrahydroxyflavone)	$C_{15}H_{10}O_{6}$	2779		28,503,991	2,170,501	1,990,396	1,750,392	1,510,027	1,330,283	
45	33'-Di-O-methylellagic acid	$C_{16}H_{10}O_{8}$	2782		32,902,975	3140073	2,989,837	2,709,887			[1]
461	Kaempferol (34'57-Tetrahydroxyflavone)	$C_{15}H_{10}O_{6}$	2924	28,705,556		2,580,529	2,130,548	1,650,185	1,530,183	1,210,287	
471	Isorhamnetin (3'-Methoxy-34'57-tetrahydroxyflavone)	$C_{16}H_{12}O_{7}$	2976		31,505,048	3,000,280	2,830,259	2,710,245	1,640,103	1,510,026	
48	Dimethoxy-trihydroxy(iso)flavone	$C17H_{14}O_{7}$	2979		32,906,613	3,140,436	2,990,199	2,710,252			
49	33'4-Tri-O-methylellagic acid	$C_{17}H_{12}O_{8}$	3014		34,304,540	3,280,227	3,129,994	2,979,758	2,850,045		
50	Undecanedioic acid	$C_{11}H_{20}O_{4}$	3082		21,512,834	1,971,179	1,531,274				
51	33'4-Tri-O-methylflavellagic acid	$C_{17}H_{12}O_{9}$	3118		35,904,031	3,440,177	3,289,943	3,139,709	3,009,995		[2]
52	Dihydroxy-dimethoxy(iso)flavone	$C_{17}H14O_{6}$	3121		31,307,122	2,980,487	2,970,408	2,830,253	2,690,452	2,550,303	
53	Pinocembrin (57-Dihydroxyflavanone)	$C_{15}H_{12}O_{4}$	3216		25,506,573	2,130,553	1,510,023	1,450,652	1,070,124	830,125	
54	Dihydroxy-trimethoxy(iso)flavone	$C_{18}H_{16}O_{7}$	3309		34,308,178	3,280,592	3,130,359	2,980,122			
55	Dodecanedioic acid	$C_{12}H_{22}O_{4}$	3328		22,914,399	2,111,335	1,671,427				
56	Hexadecanedioic acid	$C_{16}H_{30}O_{4}$	4030		28,520,659	2,671,969	2,232,064				

Table S2. Chemical composition of *A. leiocarpus* leaves methanol extract.

No.	Name	Formula	Rt	$[M + H]^+$	$[M – H]^–$	Fragment 1	Fragment 2	Fragment 3	Fragment 4	Fragment 5	Literature
1	Quinic acid	$C_7H_{12}O_6$	120		19,105,557	1,730,443	1,270,388	1,110,438	930,331	850,280	
2	Shikimic acid	$C_7H_{10}O_5$	134		17,304,500	1,550,337	1,370,234	1,110,438	930,331	730,280	
3[1]	Gallic acid (345-Trihydroxybenzoic acid)	$C_7H_6O_5$	229		16,901,370	1,250,231	970,281	810,331	790,176	690,330	[1]
4	Gallocatechin	$C_{15}H_{14}O_7$	455		30,506,613	2,610,768	2,190,662	1,790,343	1,670,340	1,250,231	
5	Protocatechuic acid (34-Dihydroxybenzoic acid)	$C_7H_6O_4$	467		15,301,879	1,090,281	1,080,204	910,175	810,332		[1]
6	3-Hydroxybenzaldehyde	$C_7H_6O_2$	1195	12,304,461		1,050,452	950,498	810,341			
7	Punicalagin	$C_{48}H_{28}O_{30}$	1200		108,305,872	7,810,555	7,210,301	6,009,904	5,750,099	3,009,993	[2]
8	Kynurenic acid	$C_{10}H_7NO_3$	1315	19,005,042		1,620,552	1,440,446	1,160,497	890,393		
9[1]	**Catechin**	$C_{15}H_{14}O_6$	1326		28,907,121	2,450,818	2,050,503	2,030,709	1,250,231	1,090,281	
10[1]	Epigallocatechin	C15H14O7	1356		30,506,613	2,190,653	1,790,339	1,670,338	1,370,232	1,250,231	
11	Casuarinin	$C_{41}H_{28}O_{26}$	1364		93,507,906	9,170,709	7,830,712	6,330,746	3,009,999	2,750,202	
12[1]	Chlorogenic acid (3-O-Caffeoylquinic acid)	$C_{16}H_{18}O_9$	1418	35,510,291		1,630,393	1,450,287	1,350,445	1,170,338	890,393	
13	Caffeic acid	$C_9H_8O_4$	1427		17,903,444	1,350,440	1,070,487				
14	Cornusiin B or isomer	$C_{48}H_{30}O_{30}$	1452		108,507,437	9,330,726	7,830,696	6,310,592	4,509,947	3,009,993	
15	Ampelopsin (Dihydromyricetin)	$C_{15}H_{12}O_8$	1464		31,904,540	3,010,356	1,930,136	1,789,978	1,530,184	1,250,231	
16	Tellimagrandin I or isomer	$C_{34}H_{26}O_{22}$	1505		78,508,375	6,330,756	6,150,632	4,830,762	3,009,993	2,750,204	
17[1]	Epigallocatechin-3-O-gallate (Teatannin II)	$C_{22}H_{18}O_{11}$	1638		45,707,709	3,310,474	3,050,672	2,870,570	1,690,133	1,250,231	
18	Coumaroylquinic acid	$C_{16}H_{18}O_8$	1680		33,709,235	1,910,556	1,730,445	1,630,390	1,190,489	930,331	
19[1]	Epicatechin	$C_{15}H_{14}O_6$	1704		28,907,121	2,450,829	2,050,499	2,030,710	1,250,230	1,090,280	
20	Corilagin or isomer	$C_{27}H_{22}O_{18}$	1749		63,307,279	4,630,533	4,190,633	3,009,994	2,750,203	1,690,134	
21	Caffeoylshikimic acid	$C_{16}H_{16}O_8$	1795		33,507,670	1,790,342	1,730,442	1,610,233	1,350,440	1,110,438	
22[1]	Taxifolin (Dihydroquercetin)	$C_{15}H_{12}O_7$	1924		30,305,048	2,850,409	1,990,398	1,750,393	1,530,184	1,250,231	
23	Ferulic acid	$C_{10}H_{10}O_4$	1925		19,305,009	1,780,262	1,490,598	1,370,233	1,340,362	1,210,280	
24[1]	Epicatechin-3-O-gallate	$C_{22}H_{18}O_{10}$	1936		44,108,218	2,890,724	2,710,615	2,450,818	1,690,132	1,250,231	
25	Chebulagic acid	$C_{41}H_{30}O_{27}$	1962		95,308,963	9,350,836	7,830,661	4,630,526	3,009,993	2,750,203	[1]
26	Ellagic acid O-hexoside isomer 1	$C_{20}H_{16}O_{13}$	1990		46,305,127	3,009,993	2,999,916				
27	Ellagic acid O-hexoside isomer 2	$C_{20}H_{16}O_{13}$	2024		46,305,127	3,009,993	2,999,915				
28	Coumaroylshikimic acid	$C_{16}H_{16}O_7$	2034		31,908,178	1,730,441	1,630,390	1,550,340	1,190,489	1,110,438	
29	Myricetin-O-hexoside	$C_{21}H_{20}O_{13}$	2089		47,908,257	3,170,305	3,160,227	2,870,201	2,710,252	2,420,219	
30	Quercetin-O-galloylhexoside	$C_{28}H_{24}O_{16}$	2156		61,509,862	4,630,889	3,130,577	3,010,356	3,000,279	2,710,248	
31	Theaflavin or isomer	$C_{29}H_{24}O_{12}$	2162	56,513,461		4,271,029	4,090,920	2,710,598	2,590,608	1,390,393	

32	Aromadendrin (Dihydrokaempferol)	$C_{15}H_{12}O_6$	2186		28,705,557	2,590,612	2,430,665	2,010,558	1,770,548	1,250,231	
33	33'-Di-O-methylellagic acid-4-O-glucoside	$C_{22}H_{20}O_{13}$	2216		49,108,257	4,760,600	3,280,229	3,129,995	2,979,758		
34	Coatline A isomer	$C_{21}H_{24}O_{10}$	2262		43,512,913	4,171,191	3,450,985	3,270,880	3,150,880	2,090,452	
35	Quercetin-3-O-glucuronide	$C_{21}H_{18}O_{13}$	2268		47,706,692	3,010,357	2,550,301	1,789,978	1,510,026		
36[1]	Isoquercitrin (Hirsutrin Quercetin-3-O-glucoside)	$C_{21}H_{20}O_{12}$	2289		46,308,765	3,010,358	3,000,280	2,710,253	1,789,978	1,510,026	
37[1]	Rutin (Quercetin-3-O-rutinoside)	$C_{27}H_{30}O_{16}$	2296	61,116,122		4,651,045	3,030,505	1,290,551	850,291		
38	Eschweilenol C (Ellagic acid-4-O-rhamnoside)	$C_{20}H_{16}O_{12}$	2308		44,705,636	3,009,994	2,999,916				[2]
39	Reinutrin (Quercetin-3-O-xyloside)	$C_{20}H_{18}O_{11}$	2320		433,07,709	3,010,353	3,000,279	2,710,253	1,789,980	1,510,023	
40	Ellagic acid	$C_{14}H_6O_8$	2335		30,099,845	2,839,964	2,570,097	2,450,094	2,290,140	1,850,239	[2]
41	Avicularin (Quercetin-3-O-arabinoside)	$C_{20}H_{18}O_{11}$	2348		43,307,709	3,010,353	3,000,279	27,10,254	1,789,976	1,510,026	
42[1]	Myricetin (33'4'55'7-Hexahydroxyflavone)	$C_{15}H_{10}O_8$	2415		31,702,974	1,920,052	1,789,979	1,650,186	1,510,026	1,370,232	
43	Guaijaverin (Quercetin-3-O-arabinoside)	$C_{20}H_{18}O_{11}$	2421		43,307,709	3,010,357	3,000,281	2,710,252	1,789,974	1,510,028	
44	Isorhamnetin-O-glucuronide isomer 1	$C_{22}H_{20}O_{13}$	2440		49,108,257	3,150,515	3,010,355	3,000,281	2,710,252	15,10,026	
45[1]	Quercitrin (Quercetin-3-O-rhamnoside)	$C_{21}H_{20}O_{11}$	2443		44,709,274	3,010,357	3,000,279	2,710,253	1,789,979	1,510,026	
46	Eriodictyol	$C_{15}H_{12}O_6$	2485		28,705,557	1,510,026	1,350,441	1,070,125			
47[1]	Isorhamnetin-3-O-glucoside	$C_{22}H_{22}O_{12}$	2491		47,710,330	3,150,516	3,140,438	2,850,409	2,710,252	2,570,457	
48	Dimethoxy-tetrahydroxy(iso)flavone-O-hexoside	$C_{23}H_{24}O_{13}$	2497		50,711,387	3,440,536	3,290,306	3160599	2,730,411	2,420,221	
49	Isorhamnetin-O-glucuronide isomer 2	$C_{22}H_{20}O_{13}$	2513		49,108,257	3,150,515	3,000,278	2710256	1,510,031		
50	Isorhamnetin-3-O-rutinoside (Narcissin)	$C_{28}H_{32}O_{16}$	2519		62,316,122	3,150,516	3,140435	3000282	2,990,201	2,710,257	
51	Di-O-methylellagic acid-O-pentoside	$C_{21}H_{18}O_{12}$	2532		46,107,200	3,280,230	3,129,997	2979757	2,850,047		
52	33'4-Tri-O-methylflavellagic acid-4-O-glucoside	$C_{23}H_{22}O_{14}$	2556		52,109,314	5,060,733	4,910,476	3580349	3,430,100	3,279,868	[3]
53	3-O-Methylellagic acid	$C_{15}H_8O_8$	2568		31,501,410	2,999,919	2,440,008	2280053			
54[1]	Quercetin	$C_{15}H_{10}O_7$	2695		30,103,483	2730,409	2,450,452	1789978	1,510,026	1,210,282	
55[1]	Naringenin	$C_{15}H_{12}O_5$	2717		27,106,065	2,270,712	1,770,184	1510026	1,190,489	1,070,125	
56[1]	Luteolin (3'4'57-Tetrahydroxyflavone)	$C_{15}H_{10}O_6$	2781		28,503,991	2,170,501	1,990,398	1750394	1,510,026	1,330,283	
57	33'-Di-O-methylellagic acid	$C_{16}H_{10}O_8$	2785		32,902,975	3,140,074	2989838	2709888			[1]
58[1]	Kaempferol (34'57-Tetrahydroxyflavone)	$C_{15}H_{10}O_6$	2926	28,705,556		2,580,529	2,130,549	1650188	1,530,182	1,210,287	
59[1]	Apigenin (4'57-Trihydroxyflavone)	$C_{15}H_{10}O_5$	2963		26,904,500	2,250,544	1,510,025	1490232	1,170,332		
60[1]	Isorhamnetin (3'-Methoxy-34'57-tetrahydroxyflavone)	$C_{16}H_{12}O_7$	2980		31,505,048	3,000,280	2,830,260	2710252	1,640,104	1,510,025	
61	Dimethoxy-trihydroxy(iso)flavone	$C_{17}H_{14}O_7$	2981		32,906,613	3,140,440	2,990,197	2710248			
62	33'4-Tri-O-methylellagic acid	$C_{17}H_{12}O_8$	3019		34,304,540	3,280,229	3,129,996	2979759	2,850,045		
63	Undecanedioic acid	$C_{11}H_{20}O_4$	3085		21,512,834	1,971,179	1,531,275				
64	33'4-Tri-O-methylflavellagic acid	$C_{17}H_{12}O_9$	3121		35,904,031	3,440,176	3,289,943	3,139,709	3,009,995		[2]

65	Dihydroxy-dimethoxy(iso)flavone	$C_{17}H_{14}O_6$	3124		31,307,122	2,980,492	2,970,404	2,830,253	2,690,427	2,550,292	
66	Pinocembrin (57-Dihydroxyflavanone)	$C_{15}H_{12}O_4$	3217		25,506,573	2,130,554	1,510,026	1,450,652	1,070,126	830,126	
67	Dihydroxy-trimethoxy(iso)flavone	$C_{18}H_{16}O_7$	3310		34,308,178	3,280,592	3,130,360	2,980,123			
68	Dodecanedioic acid	$C_{12}H_{22}O_4$	3329		22,914,399	2,111,330	1,671,430				
69	Hexadecanedioic acid	$C_{16}H_{30}O_4$	4030		28,520,659	2,671,972	2,232,062				

Table S3. Chemical composition of *A. leiocarpus* leaves water extract.

No.	Name	Formula	Rt	$[M + H]^+$	$[M - H]^-$	Fragment 1	Fragment 2	Fragment 3	Fragment 4	Fragment 5	Literature
1	Quinic acid	$C_7H_{12}O_6$	122		19,105,557	1,730,448	1,270,390	1,110,438	930,331	850,280	
2	Shikimic acid	$C_7H_{10}O_5$	134		17,304,500	1,550,335	1,370,233	1,110,437	930,331	730,279	
3[1]	Gallic acid (345-Trihydroxybenzoic acid)	$C_7H_6O_5$	229		16,901,370	1,250,231	970,281	810,330	790,176	690,331	[1]
4	Gallocatechin	$C_{15}H_{14}O_7$	445		30,506,613	2,610,768	2,190,659	1,790,342	1,670,340	1,250,231	
5	Protocatechuic acid (34-Dihydroxybenzoic acid)	$C_7H_6O_4$	466		15,301,879	1,090,281	1,080,203	910,175	810,332		Chaabi et al. 2008)
6	3-Hydroxybenzaldehyde	$C_7H_6O_2$	1198	12,304,461		1,050,456	950,498	810,342			
7	Punicalagin	$C_{48}H_{28}O_{30}$	1196		108,305,872	7,810,541	7,210,304	6,009,904	5,750,100	3,009,994	[2]
8	Kynurenic acid	$C_{10}H_7NO_3$	1307	19,005,042		1,620,552	1,440,450	1,160,499	890,392		
9[1]	**Catechin**	$C_{15}H_{14}O_6$	1328		28,907,121	2,450,819	2,050,502	2,030,710	1,250,232	1,090,281	
10[1]	Epigallocatechin	$C_{15}H_{14}O_7$	1358		30,506,613	2,190,650	1,790,338	1,670,333	1,370,232	1,250,231	
11	Casuarinin	$C_{41}H_{28}O_{26}$	1365		93,507,906	9,170,776	7,830,676	6,330,745	3,010,001	2,750,203	
12[1]	Chlorogenic acid (3-O-Caffeoylquinic acid)	$C_{16}H_{18}O_9$	1420	35,510,291		1,630,392	1,450,288	1,350,442	1,170,337	890,390	
13	Caffeic acid	$C_9H_8O_4$	1432		17,903,444	1,350,440	1,070,492				
14	Cornusiin B or isomer	$C_{48}H_{30}O_{30}$	1455		108,507,437	9,330,710	7,830,701	6,310,585	4,509,947	3,009,993	
15	Ampelopsin (Dihydromyricetin)	$C_{15}H_{12}O_8$	1467		31,904,540	3,010,352	1,930,135	1,789,974	1,530,180	1,250,231	
16	Tellimagrandin I or isomer	$C_{34}H_{26}O_{22}$	1508		78,508,375	6,330,732	6,150,657	4,830,790	3,009,993	2,750,203	
17[1]	Epigallocatechin-3-O-gallate (Teatannin II)	$C_{22}H_{18}O_{11}$	1637		45,707,709	3,310,474	3,050,676	2,870,570	1,690,132	1,250,231	
18	Coumaroylquinic acid	$C_{16}H_{18}O_8$	1682		33,709,235	1,910,555	1,730,445	1,630,389	1,190,488	930,330	
19[1]	Epicatechin	$C_{15}H_{14}O_6$	1704		28,907,121	2,450,814	2,050,501	2,030,710	1,250,230	1,090,283	
20	Corilagin or isomer	$C_{27}H_{22}O_{18}$	1749		63,307,279	4,630,527	4,190,619	3,009,993	2,750,202	1,690,133	
21	Caffeoylshikimic acid	$C_{16}H_{16}O_8$	1794		33,507,670	1,790,342	1,730,443	1,610,233	1,350,440	1,110,437	
22[1]	Taxifolin (Dihydroquercetin)	$C_{15}H_{12}O_7$	1923		30,305,048	2,850,407	1,990,395	1,750,392	1,530,182	1,250,230	
23	Ferulic acid	$C_{10}H_{10}O_4$	1925		19,305,009	1,780,261	1,490,594	1,370,230	1,340,362	1,210,281	
24[1]	Epicatechin-3-O-gallate	$C_{22}H_{18}O_{10}$	1935		44,108,218	2,890,724	2,710,622	2,450,820	1,690,132	1,250,231	
25	Chebulagic acid	$C_{41}H_{30}O_{27}$	1961		95,308,963	9,350,847	7,830,749	4,630,515	3,009,993	2,750,201	[1]
26	Ellagic acid O-hexoside isomer 1	$C_{20}H_{16}O_{13}$	1988		46,305,127	3,009,993	2,999,915				

27	Ellagic acid O-hexoside isomer 2	$C_{20}H_{16}O_{13}$	2022		46,305,127	3,009,995	2,999,916					
28	Coumaroylshikimic acid	$C_{16}H_{16}O_{7}$	2032		31,908,178	1,730,447	1,630,390	1,550,339	1,190,489	1,110,438		
29	Myricetin-O-hexoside	$C_{21}H_{20}O_{13}$	2089		47,908,257	3,170,305	3,160,226	2,870,201	2,710,252	2,420,222		
30	Quercetin-O-galloylhexoside	$C_{28}H_{24}O_{16}$	2156		61,509,862	4,630,888	3,130,571	3,010,357	3,000,279	2,710,248		
31	Aromadendrin (Dihydrokaempferol)	$C_{15}H_{12}O_{6}$	2184		28,705,557	2,590,614	2,430,665	2,010,558	1,770,546	1,250,232		
32	33'-Di-O-methylellagic acid-4-O-glucoside	$C_{22}H_{20}O_{13}$	2214		49,108,257	4,760,600	3,280,227	3,129,995	2,979,760			
33	Coatline A isomer	$C_{21}H_{24}O_{10}$	2261		43,512,913	4,171,191	3,450,984	3,270,880	3,150,877	2,090,449		
34	Quercetin-3-O-glucuronide	$C_{21}H_{18}O_{13}$	2266		47,706,692	3,010,357	2,550,301	1,789,979	1,510,026			
35[1]	Isoquercitrin (Hirsutrin Quercetin-3-O-glucoside)	$C_{21}H_{20}O_{12}$	2288		46,308,765	3,010,358	3,000,279	2,710,251	1,789,975	1,510,026		
36[1]	Rutin (Quercetin-3-O-rutinoside)	$C_{27}H_{30}O_{16}$	2298	61,116,122		4,651,022	3,030,504	1,290,550	850,291			
37	Eschweilenol C (Ellagic acid-4-O-rhamnoside)	$C_{20}H_{16}O_{12}$	2307		44,705,636	3,009,993	2,999,915					[2]
38	Reinutrin (Quercetin-3-O-xyloside)	$C_{20}H_{18}O_{11}$	2318		43,307,709	3,010,353	3,000,280	2,710,255	1,7899,76	1,510,024		
39	Ellagic acid	$C_{14}H_{6}O_{8}$	2334		30,099,845	2,839,969	2,570,094	2,450,086	2,290,140	1,850,237		[2]
40	Avicularin (Quercetin-3-O-arabinoside)	$C_{20}H_{18}O_{11}$	2347		43,307,709	3,010,355	3,000,279	2,710,250	1,789,972	1,510,024		
41[1]	Myricetin (33'4'55'7-Hexahydroxyflavone)	$C_{15}H_{10}O_{8}$	2415		31,702,974	1,920,052	1,789,976	1,650,186	1,510,026	1,370,231		
42	Guaijaverin (Quercetin-3-O-arabinoside)	$C_{20}H_{18}O_{11}$	2421		43,307,709	3,010,355	3,000,278	2,710,252	1,789975	1,510,027		
43[1]	Quercitrin (Quercetin-3-O-rhamnoside)	$C_{21}H_{20}O_{11}$	2442		44,709,274	3,010,354	3,000,276	2,710,251	1,789,975	1,510,025		
44[1]	Isorhamnetin-3-O-glucoside	$C_{22}H_{22}O_{12}$	2490		47,710,330	3,150,515	3,140,436	2,850,408	2,710,251	2,570,457		
45	Dimethoxy-tetrahydroxy(iso)flavone-O-hexoside	$C_{23}H_{24}O_{13}$	2497		50,711,387	3,440,541	3,290,306	3,160,608	2,730,406	2,420,223		
46	Isorhamnetin-O-glucuronide	$C_{22}H_{20}O_{13}$	2514		49,108,257	3,150,513	3,000,275	2,710,257	1,510,031			
47	Isorhamnetin-3-O-rutinoside (Narcissin)	$C_{28}H_{32}O_{16}$	2518		62,316,122	3,150,517	3,140,438	3,000,274	2,990,204	2,710,251		
48	Di-O-methylellagic acid-O-pentoside	$C_{21}H_{18}O_{12}$	2531		46,107,200	3,280,228	3,129,995	2,979,756	2,850,055			
49	33'4-Tri-O-methylflavellagic acid-4-O-glucoside	$C_{23}H_{22}O_{14}$	2555		52,109,314	5,060,715	4,910,467	3,580,339	3,430,098	3,279,865		[3]
50	3-O-Methylellagic acid	$C_{15}H_{8}O_{8}$	2567		31,501,410	2,999,913	2,440,008	2,280,053				
51[1]	Quercetin	$C_{15}H_{10}O_{7}$	2695		30,103,483	2,730,402	2,450,450	1,789,978	1,510,026	1,210,281		
52[1]	Naringenin	$C_{15}H_{12}O_{5}$	2716		27,106,065	2,270,712	1,770,182	1,510,026	1,190,489	1,070,125		
53[1]	Luteolin (3'4'57-Tetrahydroxyflavone)	$C_{15}H_{10}O_{6}$	2781		28,503,991	2,170,501	1,990,396	1,750,390	1,510,024	1,330,282		
54	33'-Di-O-methylellagic acid	$C_{16}H_{10}O_{8}$	2783		32,902,975	3,140,072	2,989,836	2,709,886				[1]
55[1]	Kaempferol (34'57-Tetrahydroxyflavone)	$C_{15}H_{10}O_{6}$	2929	28,705,556		2,580,529	2,130,549	1,650,188	1,530,182	1,210,287		
56[1]	Isorhamnetin (3'-Methoxy-34'57-tetrahydroxyflavone)	$C_{16}H_{12}O_{7}$	2979		31,505,048	3,000,274	2,830,260	2,710,252	1,640,104	1,510,025		

57	33'4-Tri-O-methylellagic acid	$C_{17}H_{12}O_8$	3017		34,304,540	3,280,228	3,129,995	2,979,758	2,850,049			
58	Undecanedioic acid	$C_{11}H_{20}O_4$	3084		21,512,834	1,971,176	1,531,270					
59	33'4-Tri-O-methylflavellagic acid	$C_{17}H_{12}O_9$	3121		35,904,031	3,440,176	3,289,943	3,139,709	3,009,996			[2]
60	Dihydroxy-trimethoxy(iso)flavone	$C_{18}H_{16}O_7$	3312		34,308,178	3,280,579	3,130,361	2,980,124				
61	Dodecanedioic acid	$C_{12}H_{22}O_4$	3328		22,914,399	2,111,335	1,671,428					

Table S4. Chemical composition of *A. leiocarpus* stem bark ethyl acetate extract.

No.	Name	Formula	Rt	$[M + H]^+$	$[M – H]^-$	Fragment 1	Fragment 2	Fragment 3	Fragment 4	Fragment 5	Literature
1	Quinic acid	$C_7H_{12}O_6$	122		19,105,557	1,730,446	1,270,388	1,110,438	930,331	850,280	
2	Hexahydroxydiphenoylhexose	$C_{20}H_{18}O_{14}$	123		48,106,184	4,210,427	3,009,993	2,750,202	2,570,094		
3	Shikimic acid	$C_7H_{10}O_5$	124		17,304,500	1,550,338	1,370,233	1,110,438	930,331	730,279	
4	Galloylhexose isomer 1	$C_{13}H_{16}O_{10}$	174		33,106,653	2,710,465	2,410,345	2,110,246	1,690,133	1,250,231	
5	Galloylhexose isomer 2	$C_{13}H_{16}O_{10}$	220		33,106,653	2,710,463	2,410,369	2,110,243	1,690,132	1,250,233	
6[1]	Gallic acid (345-Trihydroxybenzoic acid)	$C_7H_6O_5$	226		16,901,370	1,250,231	970,282	810,331	790,175	690,329	[1]
7	Galloylhexose isomer 3	$C_{13}H_{16}O_{10}$	281		33,106,653	2,710,462	2,410,348	2,110,243	1,690,132	1,250,230	
8	Gallocatechin	$C_{15}H_{14}O_7$	456		30,506,613	2,610,768	2,190,660	1,790,340	1,670,339	1,250,231	
9	Protocatechuic acid (34-Dihydroxybenzoic acid)	$C_7H_6O_4$	467		15,301,879	1,090,281	1,080,202	910,173	810,332		[1]
10	Procyanidin B isomer 1	$C_{30}H_{26}O_{12}$	1155		57,713,460	4,250,905	4,070,768	2,890,725	1,610,230	1,250,232	
11	Punicalagin	$C_{48}H_{28}O_{30}$	1199		108,305,872	7,810,498	7,210,322	6,009,896	5,750,081	3,009,996	[2]
12	Procyanidin B isomer 2	$C_{30}H_{26}O_{12}$	1206		57,713,460	4,250,895	4,070,771	2,890,733	1,610,230	1,250,229	
13[1]	Catechin	$C_{15}H_{14}O_6$	1326		28,907,121	2,450,820	2,050,500	2,030,709	1,250,231	1,090,281	
14[1]	Epigallocatechin	$C_{15}H_{14}O_7$	1357		30,506,613	2,610,766	2,190,658	1,790,342	1,670,340	1,250,231	
15	Casuarinin	$C_{41}H_{28}O_{26}$	1364		93,507,906	9,170,714	7,830,650	6,330,742	3,009,999	2,750,201	
16	Caffeic acid	$C_9H_8O_4$	1429		17,903,444	1,350,440	1,070,499				
17	Cornusiin B or isomer	$C_{48}H_{30}O_{30}$	1452		108,507,437	9,330,679	7,830,700	6,310,570	4,509,946	3,009,990	
18	Ampelopsin (Dihydromyricetin)	$C_{15}H_{12}O_8$	1465		31,904,540	3,010,347	1,930,138	1,789,978	1,530,177	1,250,230	
19	Tellimagrandin I or isomer	$C_{34}H_{26}O_{22}$	1502		78,508,375	6,330,761	6,150,683	4,830,796	3,009,995	2,750,203	
20	Procyanidin B isomer 3	$C_{30}H_{26}O_{12}$	1517		57,713,460	4,250,883	4,070,762	2,890,723	1,610,235	1,250,230	
21	Digalloylhexose	$C_{20}H_{20}O_{14}$	1521		48,307,749	3,310,674	3,130,570	2,710,463	1,690,132	1,250,231	
22[1]	Epigallocatechin-3-O-gallate (Teatannin II)	$C_{22}H_{18}O_{11}$	1638		45,707,709	3,310,467	3,050,671	2,870,567	1,690,132	1,250,231	
23[1]	Epicatechin	$C_{15}H_{14}O_6$	1704		28,907,121	2,450,820	2,050,499	2,030,709	1,250,230	1,090,281	
24	Punicacortein C or D	$C_{48}H_{28}O_{30}$	1716		108,305,872	10,650,468	10,470,341	7,810,509	4,489,786	3,929,890	
25	Trigalloylhexose isomer 1	$C_{27}H_{24}O_{18}$	1730		63,508,844	4,830,774	4,650,676	3,130,571	1,690,132	1,250,231	
26	Trigalloylhexose isomer 2	$C_{27}H_{24}O_{18}$	1767		63,508,844	4,830,776	4,650,674	3,130,563	1,690,131	1,250,232	
27	Di-O-methylcoruleoellagic acid	$C_{16}H_{10}O_{10}$	1796		36,101,958	3,170,306	3,020,072	2,890,358	2,740,123		
28	Mangiferin	$C_{19}H_{18}O_{11}$	1840		42,107,709	3,430,458	3,310,462	3,010,356	2,720,328	2,590,248	

29	Trigalloylhexose isomer 3	$C_{27}H_{24}O_{18}$	1901		63,508,844	4,830,753	4,650,685	3,130,562	1,690,132	1,250,231	
30[1]	Taxifolin (Dihydroquercetin)	$C_{15}H_{12}O_{7}$	1924		30,305,048	2,850,407	1,990,396	1,7503,89	1,530,174	1,250,230	
31	Tetragalloylhexose	$C_{34}H_{28}O_{22}$	1931		78,709,940	6,350,864	6,170,803	4,650,675	1,690,132	1,250,231	
32[1]	Epicatechin-3-O-gallate	$C_{22}H_{18}O_{10}$	1936		44,108,218	2,890,723	2,710,622	2,450,819	1,690,132	1,250,231	
33	Ellagic acid O-glucuronide	$C_{20}H_{14}O_{14}$	1969		47,703,054	3,009,995	2,999,917				
34	Ellagic acid O-hexoside isomer 1	$C_{20}H_{16}O_{13}$	1989		46,305,127	3,009,998	2,999,920				
35	Ellagic acid O-hexoside isomer 2	$C_{20}H_{16}O_{13}$	2022		46,305,127	3,009,996	2,999,918				
36	O-Methylellagic acid O-hexoside isomer 1	$C_{21}H_{18}O_{13}$	2037		47,706,692	3,150,162	3,140,072	3,009,981	2,989,836	2,709,886	
37	Ellagic acid C-hexoside isomer 1	$C_{20}H_{16}O_{13}$	2080	46,506,692		4,470,584	4,110,357	3,450,244	3,270,147	2,990,192	
38	Myricetin-O-hexoside	$C_{21}H_{20}O_{13}$	2090		47,908,257	3,170,298	3,160,224	2,870,194	2,710,248	2,420,219	
39	Vitexin (Apigenin-8-C-glucoside)	$C_{21}H_{20}O_{10}$	2133	43,311,348		4,151,028	3,970,930	3,370,719	3,130,713	2,830,606	
40	Pentagalloylhexose	$C_{41}H_{32}O_{26}$	2137		93,911,036	7,690,908	6,170,724	4,470,565	1,690,127	1,250,233	
41	Di-O-methylflavellagic acid O-hexoside	$C_{22}H_{20}O_{14}$	2148		50,707,749	3,440,175	3,289,941	3,139,709	2,859,756		
42	O-Methylellagic acid O-hexoside isomer 2	$C_{21}H_{18}O_{13}$	2178		47,706,692	3,150,151	3,009,986	2,999,917	2,989,847	2,709,889	
43	Aromadendrin (Dihydrokaempferol)	$C_{15}H_{12}O_{6}$	2185		28,705,557	2,590,613	2,430,667	2,010,549	1,770,551	1,250,231	
44	33'-Di-O-methylellagic acid-4-O-glucoside	$C_{22}H_{20}O_{13}$	2215		49,108,257	4,760,600	3,280,227	3,129,995	2,979,757		
45	Isovitexin (Apigenin-6-C-glucoside)	$C_{21}H_{20}O_{10}$	2225	43,311,348		4,151,018	3,970,931	3,370,710	3,130,711	2,830,604	
46	Coatline A isomer	$C_{21}H_{24}O_{10}$	2262		43,512,913	4,171,190	3,450,975	3,270,866	3,150,879	2,090,442	
47	Ellagic acid O-pentoside	$C_{19}H_{14}O_{12}$	2276		43,304,071	3,009,993	2,999,915				
48	Ellagic acid C-hexoside isomer 2	$C_{20}H_{16}O_{13}$	2306	46,506,692		4,470,553	4,110,359	3,450,246	3,270,143	2,990,197	
49	Eschweilenol C (Ellagic acid-4-O-rhamnoside)	$C_{20}H_{16}O_{12}$	2309		44,705,636	3,009,993	2,999,915				[2]
50	Ellagic acid	$C_{14}H_{6}O_{8}$	2336		30,099,845	2,839,966	2,570,092	2,450,101	2,290,137	1,850,238	[2]
51[1]	Myricetin (33'4'55'7-Hexahydroxyflavone)	$C_{15}H_{10}O_{8}$	2414		31,702,974	1,920,054	1,789,977	1,650,178	1,510,026	1,370,235	
52	Di-O-methylflavellagic acid O-pentoside	$C_{21}H_{18}O_{13}$	2470		47,706,692	4,620,466	3,440,177	3,289,943	3,139,711	2,859,770	
53	Ducheside A (3-O-Methylellagic acid-4'-O-xyloside)	$C_{20}H_{16}O_{12}$	2474		44,705,636	3,150,152	3,140,077	2,999,916	2,989,839	2,709,887	
54	Eriodictyol	$C_{15}H_{12}O_{6}$	2484		28,705,557	1,510,025	1,350,440	1,070,124			
55	Di-O-methylellagic acid-O-pentoside	$C_{21}H_{18}O_{12}$	2529		46,107,200	3,280,227	3,129,995	2,979,758	2,850,048		
56	33'4-Tri-O-methylflavellagic acid-4-O-glucoside	$C_{23}H_{22}O_{14}$	2559		52,109,314	5,060,701	4,910,471	3,580,331	3,430,098	3,279,865	[3]
57	3-O-Methylellagic acid	$C_{15}H_{8}O_{8}$	2565		31,501,410	2,999,915	2,440,015	2,280,052			
58	Di-O-methylellagic acid-O-deoxyhexoside	$C_{22}H_{20}O_{12}$	2627		47,508,766	4,600,665	3,280,233	3,129,995	2,979,757		
59[1]	Naringenin	$C_{15}H_{12}O_{5}$	2717		27,106,065	2,270,712	1,770,193	1,510,027	1,190,489	1,070,124	

60	Di-O-methylflavellagic acid isomer 1	$C_{16}H_{10}O_9$	2725		34,502,466	3,300,021	3,289,934	3,149,788	2,869,837			
61	33'-Di-O-methylellagic acid	$C_{16}H_{10}O_8$	2783		32,902,975	3,140,073	2,989,837	2,709,888			[1]	
62	Di-O-methylflavellagic acid isomer 2	$C_{16}H_{10}O_9$	2883		34,502,466	3,300,020	3,289,946	3,149,787	2,869,836			
63	Tetra-O-methylflavellagic acid isomer 1	$C_{18}H_{14}O_9$	2935		37,305,596	3,580,331	3,430,096	3,279,861	2,999,915			
64	33'4-Tri-O-methylellagic acid	$C_{17}H_{12}O_8$	3017		34,304,540	3,280,227	3,129,994	2,979,756	2,850,043			
65	Tetra-O-methylflavellagic acid isomer 2	$C_{18}H_{14}O_9$	3028		37,305,596	3,580,334	3,430,096	3,279,863	2,999,912			
66	33'4-Tri-O-methylflavellagic acid	$C_{17}H_{12}O_9$	3124		35,904,031	3,4401,76	3,289,942	3,139,709	3,009,995		[2]	

Table S5. Chemical composition of *A. leiocarpus* stem bark methanol extract.

No.	Name	Formula	Rt	$[M + H]^+$	$[M – H]^–$	Fragment 1	Fragment 2	Fragment 3	Fragment 4	Fragment 5	Literature
1	Quinic acid	$C_7H_{12}O_6$	119		19,105,557	1,730,449	1,270,389	1,110,438	930,330	850,280	
2	Hexahydroxydiphenoylhexose	$C_{20}H_{18}O_{14}$	122		48,106,184	4,210,429	3,009,993	2,750,202	2,570,092		
3	Shikimic acid	$C_7H_{10}O_5$	132		17,304,500	1,550,339	1,370,232	1,110,438	930,331	730,280	
4	Galloylquinic acid isomer 1	$C_{14}H_{16}O_{10}$	152		34,306,653	1,910,555	1,690,132	1,250,230			
5	Galloylhexose isomer 1	$C_{13}H_{16}O_{10}$	170		33,106,653	2,710,464	2,4103,56	2,110,243	1,690,132	1,250,231	
6	Galloylhexose isomer 2	$C_{13}H_{16}O_{10}$	219		33,106,653	2,710,463	2,410,353	2,110,244	1,690,132	1,250,231	
7[1]	Gallic acid (345-Trihydroxybenzoic acid)	$C_7H_6O_5$	224		16,901,370	1,250,231	9,702,80	810,334	790,176	690,330	[1]
8	Galloylhexose isomer 3	$C_{13}H_{16}O_{10}$	282		33,106,653	2,710,463	241,0345	2,110,243	1,690,132	1,250,230	
9	Galloylquinic acid isomer 2	$C_{14}H_{16}O_{10}$	297		34,306,653	1,910,553	1,690,133	1,250,231			
10	Galloylquinic acid isomer 3	$C_{14}H_{16}O_{10}$	369		34,306,653	1,910,552	1,690,133	1,250,231			
11	Gallocatechin	$C_{15}H_{14}O_7$	453		30,506,613	2,610,769	2,190,658	1,790,342	1,670,340	1,250,231	
12	Protocatechuic acid (34-Dihydroxybenzoic acid)	$C_7H_6O_4$	464		15,301,879	1,090,281	1,080,203	910,173	810,332		[1]
13	Procyanidin B isomer 1	$C_{30}H_{26}O_{12}$	1157		57,713,460	4,250,881	4,070,776	2,890,724	1,610,234	1,250,230	
14	Punicalagin	$C_{48}H_{28}O_{30}$	1199		108,305,872	7,810,530	7,210,322	6,009,894	5,750,092	3,009,995	[2]
15	Procyanidin B isomer 2	$C_{30}H_{26}O_{12}$	1203		57,713,460	4,250,865	4,070,771	2,890,724	1,610,241	1,250,232	
16[1]	Catechin	$C_{15}H_{14}O_6$	1327		28,907,121	2,450,819	2,050,503	2,030,709	1,250,231	1,090,281	
17[1]	Epigallocatechin	$C_{15}H_{14}O_7$	1358		30,506,613	2,610,769	2,190,656	1,790,341	1,670,339	1,250,231	
18	Casuarinin	$C_{41}H_{28}O_{26}$	1364		93,507,906	9,170,715	7,830,659	6,330,742	3,009,999	2,750,201	
19	Caffeic acid	$C_9H_8O_4$	1430		17,903,444	1,350,440	1,070,488				
20	Cornusiin B or isomer	$C_{48}H_{30}O_{30}$	1453		108,507,437	9,330,726	7,830,691	6,310,604	4,509,947	3,009,993	
21	Ampelopsin (Dihydromyricetin)	$C_{15}H_{12}O_8$	1467		31,904,540	3,010,349	1,930,137	1,789,978	1,530,181	1,250,229	
22	Tellimagrandin I or isomer	$C_{34}H_{26}O_{22}$	1506		78,508,375	6,330,737	6,150,683	4,830,819	3,009,993	2,750,204	
23	Procyanidin B isomer 3	$C_{30}H_{26}O_{12}$	1517		57,713,460	4,250,886	4,070,778	2,890,721	1,610,232	1,250,231	
24	Digalloylhexose	$C_{20}H_{20}O_{14}$	1523		48,307,749	3,310,680	3,130,569	2,710,462	1,690,132	1,250,231	
25[1]	Epigallocatechin-3-O-gallate (Teatannin II)	$C_{22}H_{18}O_{11}$	1636		45,707,709	3,310,465	3,050,667	2,870,563	1,690,132	1,250,231	
26[1]	Epicatechin	$C_{15}H_{14}O_6$	1704		28,907,121	2,450,818	2,050,501	2,030,708	1,250,231	1,090,281	
27	Punicacortein C or D	$C_{48}H_{28}O_{30}$	1715		108,305,872	10,650,499	10,470,348	7,810,545	4,489,790	3,929,892	
28	Trigalloylhexose isomer 1	$C_{27}H_{24}O_{18}$	1729		63,508,844	4,830,786	4,650,676	3,130,571	1,690,132	1,250,231	
29	Trigalloylhexose isomer 2	$C_{27}H_{24}O_{18}$	1768		63,508,844	4,830,776	4,650,668	3,130,567	1,690,132	1,250,230	
30	Mangiferin	$C_{19}H_{18}O_{11}$	1839		42,107,709	3,430,462	3,310,462	3,010,356	2,720,328	2,590,249	
31	Trigalloylhexose isomer 3	$C_{27}H_{24}O_{18}$	1901		63,508,844	4,830,776	4,650,669	3,130,573	1,690,132	1,250,230	

32[1]	Taxifolin (Dihydroquercetin)	$C_{15}H_{12}O_{7}$	1924		30,305,048	2,850,409	1,990,396	1,750,390	1,530,188	1,250,231	
33	Tetragalloylhexose	$C_{34}H_{28}O_{22}$	1930		78,709,940	6,350,890	6,170,790	4,650,675	1,690,132	1,250,231	
34[1]	Epicatechin-3-O-gallate	$C_{22}H_{18}O_{10}$	1935		44,108,218	2,890,721	2,710,614	2,450,819	1,690,134	1,250,231	
35	Ellagic acid O-glucuronide	$C_{20}H_{14}O_{14}$	1967		47,703,054	3,009,993	2,999,915				
36	Ellagic acid O-hexoside isomer 1	$C_{20}H_{16}O_{13}$	1990		46,305,127	3,009,993	2,999,914				
37	Ellagic acid O-hexoside isomer 2	$C_{20}H_{16}O_{13}$	2021		46,305,127	3,009,995	2,999,915				
38	O-Methylellagic acid O-hexoside isomer 1	$C_{21}H_{18}O_{13}$	2037		47,706,692	3,150,173	3,140,074	3,009,994	2,989,837	2,709,890	
39	Ellagic acid C-hexoside isomer 1	$C_{20}H_{16}O_{13}$	2079	46,506,692		4,470,584	4,110,357	3,450,244	3,270,147	2,990,192	
40	Myricetin-O-hexoside	$C_{21}H_{20}O_{13}$	2090		47,908,257	3,170,309	3,160,227	2,870,202	2,710,249	2,420,219	
41	Vitexin (Apigenin-8-C-glucoside)	$C_{21}H_{20}O_{10}$	2134	43,311,348		4,151,028	397,0917	3,370,724	3,130,711	2,830,606	
42	Pentagalloylhexose	$C_{41}H_{32}O_{26}$	2139		93,911,036	7,690,908	6,170,821	4,470,565	1,690,133	1,250,230	
43	Di-O-methylflavellagic acid O-hexoside	$C_{22}H_{20}O_{14}$	2149		50,707,749	3,440,176	3,289,942	3,139,709	2,859,758		
44	Theaflavin or isomer	$C_{29}H_{24}O_{12}$	2162	56,513,461		4,271,026	4,090,923	2,710,598	2,590,605	1,390,392	
45	O-Methylellagic acid O-hexoside isomer 2	$C_{21}H_{18}O_{13}$	2179		47,706,692	3,150,152	3,009,992	2,999,915	2,989,837	2,709,872	
46	Aromadendrin (Dihydrokaempferol)	$C_{15}H_{12}O_{6}$	2185		28,705,557	2,590,617	2,430,667	2,010,549	1,770,551	1,250,231	
47	33'-Di-O-methylellagic acid-4-O-glucoside	$C_{22}H_{20}O_{13}$	2214		49,108,257	4,760,603	3,280,229	3,129,995	2,979,758		
48	Isovitexin (Apigenin-6-C-glucoside)	$C_{21}H_{20}O_{10}$	2225	43,311,348		4,151,042	3,970,922	3,370,714	3,130,713	2,830,606	
49	Coatline A isomer	$C_{21}H_{24}O_{10}$	2262		43,512,913	4,171,191	3,450,985	3,270,863	3,150,880	2,090,455	
50	Ellagic acid O-pentoside	$C_{19}H_{14}O_{12}$	2276		43,304,071	3,009,993	2,999,916				
51	Ellagic acid C-hexoside isomer 2	$C_{20}H_{16}O_{13}$	2303	46,506,692		4,470,553	4,110,359	3,450,246	3,270,143	2,990,197	
52	Eschweilenol C (Ellagic acid-4-O-rhamnoside)	$C_{20}H_{16}O_{12}$	2309		44,705,636	3,009,993	2,999,915				[2]
53	Ellagic acid	$C_{14}H_{6}O_{8}$	2336		30,099,845	2,839,969	2,570,091	2,450,093	2,290,140	1,850,238	[2]
54[1]	Myricetin (33'4'55'7-Hexahydroxyflavone)	$C_{15}H_{10}O_{8}$	2416		31,702,974	1,920,052	1,789,977	1,650,180	1,510,026	1,370,231	
55	Ducheside A (3-O-Methylellagic acid-4'-O-xyloside)	$C_{20}H_{16}O_{12}$	2475		44,705,636	3,150,152	3,140,072	2,999,914	2,989,838	2,709,881	
56	Eriodictyol	$C_{15}H_{12}O_{6}$	2485		28,705,557	1,510,025	1,350,440	1,070,124			
57	4-Methoxycinnamic acid	$C_{10}H_{10}O_{3}$	2528		17,907,082	1,610,600	1,330,652	1,050,704	1,030,543	790,545	
58	Di-O-methylellagic acid-O-pentoside	$C_{21}H_{18}O_{12}$	2533		46,107,200	3,280,227	3,129,995	2,979,758	2,850,045		
59	33'4-Tri-O-methylflavellagic acid-4-O-glucoside	$C_{23}H_{22}O_{14}$	2561		52,109,314	5,060,700	4,910,468	3,580,332	3,430,098	3,279,864	[3]
60	3-O-Methylellagic acid	$C_{15}H_{8}O_{8}$	2564		31,501,410	2,999,915	2,440,010	2,280,052			
61	Di-O-methylellagic acid-O-deoxyhexoside	$C_{22}H_{20}O_{12}$	2627		47,508,766	4,600,647	3,280,225	3,129,995	2,979,759		
62[1]	Naringenin	$C_{15}H_{12}O_{5}$	2717		27,106,065	2,270,712	1,770,193	1,510,026	1,190,489	1,070,125	
63	Di-O-methylflavellagic acid isomer 1	$C_{16}H_{10}O_{9}$	2726		34,502,466	3,300,020	3,289,932	3,149,787	2,869,837		
64[1]	Luteolin (3'4'57-Tetrahydroxyflavone)	$C_{15}H_{10}O_{6}$	2780		28,503,991	2,170,501	1,990,398	1,750,392	1,510,024	1,330,285	
65	33'-Di-O-methylellagic acid	$C_{16}H_{10}O_{8}$	2785		32,902,975	3,140,072	2,989,836	2,709,887			[1]

66	Di-O-methylflavellagic acid isomer 2	$C_{16}H_{10}O_9$	2882		34,502,466	3,300,020	3,289,944	3,149,787	2,869,836		
67	Tetra-O-methylflavellagic acid isomer 1	$C_{18}H_{14}O_9$	2934		37,305,596	3,580,335	3,430,097	3,279,862	2,999,913		
68	33'4-Tri-O-methylellagic acid	$C_{17}H_{12}O_8$	3016		34,304,540	3,280,227	3,129,995	2,979,757	2,850,043		
69	Tetra-O-methylflavellagic acid isomer 2	$C_{18}H_{14}O_9$	3027		37,305,596	3,580,333	3,430,096	3,279,863	2,999,911		
70	33'4-Tri-O-methylflavellagic acid	$C_{17}H_{12}O_9$	3121		35,904,031	3,440,176	3,289,942	3,139,710	3,009,993		[2]
71	Pinocembrin (57-Dihydroxyflavanone)	$C_{15}H_{12}O_4$	3217		25,506,573	2,130,557	1,510,025	1,450,652	1,070,132	830,123	

Table S6. Chemical composition of *A. leiocarpus* stem bark water extract.

**No.**	**Name**	**Formula**	**Rt**	**$[M + H]^+$**	**$[M – H]^–$**	**Fragment 1**	**Fragment 2**	**Fragment 3**	**Fragment 4**	**Fragment 5**	**Literature**
1	Quinic acid	$C_7H_{12}O_6$	123		19,105,557	1,730,443	1,270,389	1,110,441	930,332	850,280	
2	Shikimic acid	$C_7H_{10}O_5$	132		17,304,500	1,550,341	1,370,233	1,110,438	930,331	730,280	
3	Hexahydroxydiphenoylhexose	$C_{20}H_{18}O_{14}$	137		48,106,184	4,210,407	3,009,994	2,750,203	2,570,093		
4	Galloylquinic acid isomer 1	$C_{14}H_{16}O_{10}$	148		34,306,653	1,910,556	1,690,133	1,250,230			
5	Galloylhexose isomer 1	$C_{13}H_{16}O_{10}$	175		33,106,653	2,710,462	2,410,351	2,110,241	1,690,133	1,250,231	
6	Galloylhexose isomer 2	$C_{13}H_{16}O_{10}$	209		33,106,653	2,710,464	2,410,348	2,110,243	1,690,132	1,250,231	
7[1]	Gallic acid (345-Trihydroxybenzoic acid)	$C_7H_6O_5$	228		16,901,370	1,250,231	970,282	810,333	790,174	690,330	[1]
8	Galloylquinic acid isomer 2	$C_{14}H_{16}O_{10}$	252		34,306,653	1,910,556	1,690,135	1,250,231			
9	Galloylhexose isomer 3	$C_{13}H_{16}O_{10}$	276		33,106,653	2,710,464	2,410,351	2,110,244	1,690,133	1,250,231	
10	Galloylquinic acid isomer 3	$C_{14}H_{16}O_{10}$	365		34,306,653	1,910,556	1,690,133	1,250,232			
11	Gallocatechin	$C_{15}H_{14}O_7$	448		30,506,613	2,610,769	2,190,659	1,790,343	1,670,339	1,250,231	
12	Protocatechuic acid (34-Dihydroxybenzoic acid)	$C_7H_6O_4$	469		15,301,879	1,090,281	1,080,203	910,176	810,330		[1]
13	Punicalagin	$C_{48}H_{28}O_{30}$	1198		108,305,872	7,810,522	7,210,322	6,009,882	5,750,122	3,009,994	[2]
14	Procyanidin B isomer 1	$C_{30}H_{26}O_{12}$	1202		57,713,460	4,250,898	4,070,774	2,890,724	1,610,233	1,250,231	
15[1]	Catechin	$C_{15}H_{14}O_6$	1329		28,907,121	2,450,819	2,050,503	2,030,709	1,250,231	1,090,281	
16[1]	Epigallocatechin	$C_{15}H_{14}O_7$	1359		30,506,613	2,610,771	2,190,658	1,790,342	1,670,340	1,250,231	
17	Casuarinin	$C_{41}H_{28}O_{26}$	1364		93,507,906	9,170,743	7,830,739	6,330,744	3,010,000	2,750,202	
18	Caffeic acid	$C_9H_8O_4$	1430		17,903,444	1,350,440	1,070,490				
19	Ampelopsin (Dihydromyricetin)	$C_{15}H_{12}O_8$	1469		31,904,540	3,010,349	1,930,138	1,789,982	1,530,185	1,250,232	
20	Tellimagrandin I or isomer	$C_{34}H_{26}O_{22}$	1507		78,508,375	6,330,803	6,150,612	4,830,777	3,009,994	2,750,205	
21	Procyanidin B isomer 2	$C_{30}H_{26}O_{12}$	1519		57,713,460	4,250,891	4,070,773	2,890,729	1,610,230	1,250,231	
22	Digalloylhexose	$C_{20}H_{20}O_{14}$	1524		48,307,749	3,310,674	3,130,571	2,710,463	1,690,132	1,250,231	
23[1]	Epigallocatechin-3-O-gallate (Teatannin II)	$C_{22}H_{18}O_{11}$	1638		45,707,709	3,310,464	3,050,671	2,870,566	1,690,133	1,250,231	
24[1]	Epicatechin	$C_{15}H_{14}O_6$	1705		28,907,121	2,450,820	2,050,502	2,030,712	1,250,231	1,090,281	
25	Punicacortein C or D	$C_{48}H_{28}O_{30}$	1716		108,305,872	10,650,501	10,470,340	7,8105,74	4,489,796	3,929,891	
26	Trigalloylhexose isomer 1	$C_{27}H_{24}O_{18}$	1732		63,508,844	4,830,768	4,650,681	3,130,573	1,690,133	1,250,231	
27	Trigalloylhexose isomer 2	$C_{27}H_{24}O_{18}$	1765		63,508,844	4,830,776	4,650,691	3,130,558	1,690,133	1,250,233	

28	Mangiferin	$C_{19}H_{18}O_{11}$	1840		42,107,709	3,430,457	3,310,463	3,010,357	2,720,330	2,590,248	
29	Trigalloylhexose isomer 3	$C_{27}H_{24}O_{18}$	1900		63,508,844	4,830,771	4,650,666	3,130,575	1,690,133	1,250,231	
30[1]	Taxifolin (Dihydroquercetin)	$C_{15}H_{12}O_{7}$	1924		30,305,048	2,850,412	1,990,396	1,750,395	1,530,185	1,250,228	
31[1]	Epicatechin-3-O-gallate	$C_{22}H_{18}O_{10}$	1936		44,108,218	2,890,722	2,710,614	2,450,822	1,690,133	1,250,231	
32	Ellagic acid O-glucuronide	$C_{20}H_{14}O_{14}$	1967		47,703,054	3,009,994	2,999,918				
33	Ellagic acid O-hexoside isomer 1	$C_{20}H_{16}O_{13}$	1990		46,305,127	3,009,994	2,999,916				
34	Ellagic acid O-hexoside isomer 2	$C_{20}H_{16}O_{13}$	2021		46,305,127	3,009,993	2,999,914				
35	O-Methylellagic acid O-hexoside isomer 1	$C_{21}H_{18}O_{13}$	2037		47,706,692	3,150,141	3,140,074	3,009,994	2,989,838	2,709,887	
36	Ellagic acid C-hexoside isomer 1	$C_{20}H_{16}O_{13}$	2079	46,506,692		4,470,571	4,110,374	3,450,248	3,270,144	2,990,193	
37	Myricetin-O-hexoside	$C_{21}H_{20}O_{13}$	2091		47,908,257	3,170,309	3,160,224	2,870,199	2,710,254	2,420,219	
38	Vitexin (Apigenin-8-C-glucoside)	$C_{21}H_{20}O_{10}$	2133	43,311,348		4,151,028	3,970,927	3,370,713	3,130,712	2,830,608	
39	Di-O-methylflavellagic acid O-hexoside	$C_{22}H_{20}O_{14}$	2149		50,707,749	3,440,176	3,289,943	3,139,710	2,859,746		
40	O-Methylellagic acid O-hexoside isomer 2	$C_{21}H_{18}O_{13}$	2180		47,706,692	3,150,152	3,009,994	2,999,914	2,989,839	2,709,879	
41	33'-Di-O-methylellagic acid-4-O-glucoside	$C_{22}H_{20}O_{13}$	2215		49,108,257	4,760,605	3,280,227	3,129,995	2,979,757		
42	Luteolin-7-O-glucoside (Cynaroside)	$C_{21}H_{20}O_{11}$	2234		44,709,274	3,270,522	2,850,410	2,840,331	1,510,026		
43	Luteolin-O-deoxyhexosylhexoside	$C_{27}H_{30}O_{15}$	2241		59,315,065	2,850,410	2,840,333	1,330,282			
44	Isovitexin (Apigenin-6-C-glucoside)	$C_{21}H_{20}O_{10}$	2225	43,311,348		4,151,028	3,970,922	3,370,712	3,130,712	2,830,605	
45	Ellagic acid O-pentoside	$C_{19}H_{14}O_{12}$	2275		43,304,071	3,009,994	2,999,917				
46	Ellagic acid C-hexoside isomer 2	$C_{20}H_{16}O_{13}$	2303	46,506,692		4,470,556	4,110,362	3,450,246	3,270,150	2,990,192	
47	Eschweilenol C (Ellagic acid-4-O-rhamnoside)	$C_{20}H_{16}O_{12}$	2308		44,705,636	3,009,994	2,999,916				[2]
48	Ellagic acid	$C_{14}H_{6}O_{8}$	2335		30,099,845	2,839,968	2,570,095	2,450,097	2,290,141	1,850,238	[2]
49[1]	Myricetin (33'4'55'7-Hexahydroxyflavone)	$C_{15}H_{10}O_{8}$	2415		31,702,974	1,920,052	1,789,984	1,650,180	1,510,028	1,370,231	
50	Ducheside A (3-O-Methylellagic acid-4'-O-xyloside)	$C_{20}H_{16}O_{12}$	2474		44,705,636	3,150,154	3,140,074	2,999,916	2,989,838	2,709,898	
51	Eriodictyol	$C_{15}H_{12}O_{6}$	2485		28,705,557	1,510,029	1,350,441	1,070,126			
52	Di-O-methylellagic acid-O-pentoside	$C_{21}H_{18}O_{12}$	2533		46,107,200	3,280,229	3,129,996	2,979,760	2,850,047		
53	33'4-Tri-O-methylflavellagic acid-4-O-glucoside	$C_{23}H_{22}O_{14}$	2559		52,109,314	5,060,698	4,910,473	3,580,338	3,430,100	3,279,865	[3]
54	3-O-Methylellagic acid	$C_{15}H_{8}O_{8}$	2565		31501410	2,999,915	2,440,009	2,280,056			
55	Di-O-methylellagic acid-O-deoxyhexoside	$C_{22}H_{20}O_{12}$	2625		47,508,766	4,600,624	3,280,230	3,129,998	2,979,758		
56[1]	Naringenin	$C_{15}H_{12}O_{5}$	2717		27,106,065	2,270,712	1,770,193	1,510,026	1,190,490	1,070,125	
57	Di-O-methylflavellagic acid isomer 1	$C_{16}H_{10}O_{9}$	2725		34,502,466	3,300,023	3,289,966	3,149,789	2,869,838		
58[1]	Luteolin (3'4'57-Tetrahydroxyflavone)	$C_{15}H_{10}O_{6}$	2780		28,503,991	2,170,487	1,990,399	1,750,392	1,510,024	1,330,282	

59	33'-Di-O-methylellagic acid	$C_{16}H_{10}O_8$	2786		32,902,975	3,140,074	2,989,838	2,709,888			[1]
60	Di-O-methylflavellagic acid isomer 2	$C_{16}H_{10}O_9$	2882		34,502,466	3,300,022	3,289,951	3,149,789	2,869,838		
61	Tetra-O-methylflavellagic acid isomer 1	$C_{18}H_{14}O_9$	2935		37,305,596	3,580,334	3,430,099	3,279,862	2,999,918		
62[1]	Apigenin (4'57-Trihydroxyflavone)	$C_{15}H_{10}O_5$	2962		26,904,500	2,250,559	1,510,027	1,490,235	1,170,332		
63	33'4-Tri-O-methylellagic acid	$C_{17}H_{12}O_8$	3016		34,304,540	3,280,230	3,129,996	2,979,759	2,850,043		
64	Tetra-O-methylflavellagic acid isomer 2	$C_{18}H_{14}O_9$	3025		37,305,596	3,580,335	3,430,100	3,279,864	2,999,920		
65	33'4-Tri-O-methylflavellagic acid	$C_{17}H_{12}O_9$	3121		35,904,031	3,440,176	3,289,944	3,139,709	3,009,998		[2]

1. Chaabi M.; Benayache S.; Benayache F.; N'Gom S.; Koné M.; Anton R.; Weniger B.; Lobstein A. Triterpenes and polyphenols from Anogeissus leiocarpus (Combretaceae). *Biochemical Systematics and Ecology* **2008** 1 (36) 59-62.
2. Shuaibu M. N.; Wuyep P. T.; Yanagi T.; Hirayama K.; Ichinose A.; Tanaka T.; Kouno I. Trypanocidal activity of extracts and compounds from the stem bark of Anogeissus leiocarpus and Terminalia avicennoides. *Parasitology research* **2008** 102 (4) 697-703.
3. Adigun J.; Amupitan J.; Kelly D. Isolation and investigation of antimicrobial effect of 3 4 3'-tri-O-methylflavellagic acid and its glucoside from Anogeissus leocarpus. *Bulletin of the Chemical Society of Ethiopia* **2000** 14 (2) 169-174.
